# Plant-Derived Bioactive Compounds in Inflammation-Related Cancers: Mechanisms and Therapeutic Potential

**DOI:** 10.3390/plants15040575

**Published:** 2026-02-12

**Authors:** Mingzhu Song, Xiaolong Zhu, Xiaohong Zhao, Jiao Feng, Xinbing Sui

**Affiliations:** School of Pharmacy, Hangzhou Normal University, Hangzhou 311121, China; 18073426604@163.com (M.S.); 2023112025090@stu.hznu.edu.cn (X.Z.); zhaoxiaohong27@163.com (X.Z.)

**Keywords:** plant-derived bioactive compounds, inflammation-cancer transformation, inflammatory signaling pathways, tumor microenvironment

## Abstract

Chronic inflammation is a well-established driving force in tumor initiation and progression, accounting for a substantial proportion of inflammation-associated malignancies. Persistent inflammatory stimulation creates a pathological microenvironment characterized by sustained inflammatory signaling, oxidative stress, immune dysregulation, and epigenetic reprogramming, which collectively promote genomic instability, malignant transformation, and tumor progression. Understanding the biological basis of inflammation–cancer transformation is therefore essential for the development of effective preventive and therapeutic strategies. Plant-derived bioactive compounds have attracted increasing attention as promising modulators of inflammation-driven carcinogenesis due to their structural diversity, multi-target regulatory capacity, and relatively low toxicity. Specifically, this review focuses on four major classes of these compounds: flavonoids, alkaloids, terpenoids, and curcuminoids. Accumulating evidence demonstrates that these compounds can effectively interrupt the inflammation–cancer continuum by simultaneously targeting multiple pathogenic processes rather than single molecular pathways. In particular, these plant-derived agents suppress inflammation-driven signaling cascades, including NF-κB, MAPK, and JAK/STAT pathways; attenuate oxidative stress and inflammation-induced DNA damage; reprogram the immune microenvironment to restore anti-tumor immunity; and modulate epigenetic and transcriptional programs that stabilize pro-tumorigenic phenotypes. Accordingly, this review synthesizes the shared pathological drivers of inflammation–cancer transformation and summarizes how plant-derived compounds collectively target these mechanisms to interrupt disease progression. In addition, emerging translational strategies, including combination therapy and nanocarrier-based delivery systems, are discussed to highlight the clinical potential of plant-derived interventions. Collectively, this review offers an integrated mechanistic framework for understanding and exploiting plant-derived bioactive compounds in the prevention and treatment of inflammation-related cancers.

## 1. Introduction

Chronic inflammation is now widely recognized as a critical driving force in tumor initiation, progression, and metastasis. Unlike acute inflammation, which is generally self-limiting and protective, persistent inflammatory stimulation creates a pathological microenvironment characterized by sustained cytokine production, oxidative stress, immune dysregulation, and aberrant tissue repair. Accumulating epidemiological and experimental evidence indicates that approximately 20% of human cancers arise in the context of long-standing inflammatory diseases, underscoring inflammation as a fundamental enabling factor in carcinogenesis [1]. The process by which chronic inflammation evolves into malignancy—referred to as inflammation–cancer transformation—is not a single linear event but a multistep pathological continuum. Persistent inflammatory stimuli promote genomic instability through reactive oxygen and nitrogen species, activate oncogenic signaling pathways, remodel the immune microenvironment, and induce epigenetic alterations that collectively drive malignant transformation. Key signaling networks, including NF-κB, MAPK, PI3K/Akt, and JAK/STAT, act as central hubs linking inflammatory cues to abnormal cell proliferation, survival, angiogenesis, immune escape, and epithelial–mesenchymal transition. Consequently, targeting inflammation-driven molecular events has emerged as a promising strategy for cancer prevention and intervention.

In recent years, increasing attention has been directed toward Traditional Chinese Medicine (TCM) as a rich source of plant-derived bioactive compounds with potential to modulate inflammation-related carcinogenesis. Characterized by a multi-component and multi-target therapeutic philosophy, TCM encompasses numerous phytochemicals with diverse chemical structures and pleiotropic biological activities, enabling simultaneous regulation of multiple inflammatory and oncogenic pathways. Compared with conventional chemotherapeutic agents, TCM-derived compounds often exhibit lower systemic toxicity and better tolerability, making them particularly suitable for long-term intervention in chronic inflammatory conditions that predispose one to cancer.

Among TCM-derived bioactive compounds, flavonoids such as baicalin and baicalein have been extensively investigated for their ability to suppress inflammatory signaling, remodel the tumor microenvironment, and inhibit tumor growth, highlighting their translational potential in inflammation-associated cancers [2]. Beyond single compounds, TCM formulas further exemplify the integrative regulatory capacity of this therapeutic system. For instance, Huang qin Tang has been shown to attenuate colitis-associated colorectal cancer by regulating amino acid metabolism and inhibiting the PI3K/AKT/mTOR signaling pathway [3]. Similarly, active constituents derived from *Diphylleia sinensis* suppress colorectal cancer progression by targeting MAPK14, upregulating Slurp1 expression, and inhibiting pro-tumorigenic macrophage polarization. These findings underscore the unique advantages of TCM in concurrently modulating inflammatory signaling, immune responses, and tumor-associated metabolic pathways [4].

Despite accumulating evidence supporting the anti-inflammatory and anti-tumor potential of TCM-derived bioactive compounds, a comprehensive and mechanistically integrated understanding of how these agents interrupt the inflammation–cancer transition remains limited. Existing studies are often fragmented, focusing on individual compounds, signaling pathways, or disease models, thereby restricting a holistic view of their shared regulatory principles. Moreover, the translation of preclinical findings into clinical application remains a major challenge, highlighting the urgent need for systematic evaluation of molecular mechanisms, therapeutic strategies, and emerging delivery technologies.

In this review, we synthesize current advances in understanding the mechanisms underlying inflammation-driven carcinogenesis and critically summarize how plant-derived bioactive compounds block this pathological transition. We focus on their regulatory roles in inflammatory signaling pathways, oxidative stress, immune microenvironment remodeling, epigenetic modulation, and combination therapeutic strategies, including nanocarrier-based delivery systems. By integrating mechanistic insights with translational perspectives, this review aims to provide a coherent framework for the rational development and clinical application of plant-derived agents in the prevention and treatment of inflammation-related cancers.

## 2. Biological Basis of Inflammation–Cancer Transformation

### 2.1. Pathological Features of Inflammation-Driven Carcinogenesis

Chronic inflammation is widely recognized as an important driver of tumorigenesis, with approximately 20% of human cancers associated with long-standing inflammatory conditions [5]. Persistent inflammation increases cancer risk and accelerates tumor progression through sustained infection or metabolic dysregulation. The process of inflammation–cancer transformation can be broadly divided into several critical stages. Phase 1, the acute inflammatory phase, represents the body’s initial immune response to infection or tissue injury. This stage often activates adaptive immunity and may even exert anti-tumor effects; however, failure to resolve acute inflammation can lead to subsequent pathological progression [6]. Phase 2, characterized by chronic inflammation and immune dysregulation, is a pivotal risk period for tumor initiation. Persistent inflammatory stimuli drive continuous release of cytokines and reactive oxygen species, excessive leukocyte infiltration, and impaired tissue repair. During this phase, chronic inflammation promotes carcinogenesis through mechanisms such as immune suppression, epithelial–mesenchymal transition (EMT) [7], and the protumorigenic activities of immune cells—including macrophages—within the inflamed microenvironment. Phase 3 involves aberrant tissue repair and fibrosis, in which long-term inflammation disrupts normal regeneration processes and increases the probability of malignant transformation [8]. Metabolic reprogramming and adaptive genetic alterations emerging in this microenvironment may further accelerate tumor development. In the final phase, tumor progression and acquisition of malignant phenotypes, chronic inflammation continues to drive oncogenic evolution, including phenotypic shifts (e.g., basal cell carcinoma transitioning to squamous cell carcinoma), immune editing and immune escape, and enhanced metastatic potential mediated by EMT and remodeling of the tumor microenvironment.

Chronic inflammation serves as a central driver of inflammation-associated tumorigenesis across multiple organ systems, and this pathogenic process follows a remarkably consistent biological paradigm. The core mechanisms underlying the inflammation–cancer transition in colorectal cancer (CRC) include persistent stimulation by a chronic inflammatory microenvironment, activation of key signaling pathways, dysfunction of immune cells (e.g., macrophages), epigenetic regulation, and the influence of the gut microbiota. Among these mediators, tumor necrosis factor-α (TNF-α) regulates myeloid cell infiltration and epithelial cell proliferation through TNF-R1 and TNF-R2, respectively, while interleukin-6 (IL-6) promotes tumor development via both classical and trans-signaling pathways. The synergistic interplay between TNF-α and IL-6 further accelerates the progression from chronic colitis to colitis-associated colorectal cancer (CAC) [9]. A comparable inflammation-driven cascade underlies malignant transformation in esophagitis. In patients with chronic esophageal inflammation, particularly those with Barrett’s esophagus, prolonged exposure of the esophageal mucosa to refluxate results in persistent epithelial injury, aberrant tissue remodeling, and increased genomic instability. As a consequence, Barrett’s esophagus markedly elevates the risk of esophageal adenocarcinoma, with most cases arising in the context of long-standing inflammatory damage [10]. Chronic pancreatitis provides another representative example in which sustained inflammatory stimulation promotes carcinogenesis. Persistent pancreatic inflammation is associated with the accumulation of oncogenic mutations, such as KRAS, and the overexpression of inflammatory mediators. Together, these alterations drive the stepwise progression from pancreatic intraepithelial neoplasia to pancreatic ductal adenocarcinoma by enhancing proliferative signaling, suppressing apoptosis, promoting angiogenesis, and exacerbating inflammation-induced DNA damage [11]. Together, these alterations drive the stepwise progression from pancreatic intraepithelial neoplasia to pancreatic ductal adenocarcinoma by enhancing proliferative signaling, suppressing apoptosis, promoting angiogenesis, and exacerbating inflammation-induced DNA damage.

Collectively, these inflammation-associated malignancies illustrate a shared mechanistic framework in which persistent inflammatory injury disrupts epithelial integrity, reshapes cytokine signaling networks, and establishes a tumor-promoting microenvironment. This common paradigm underscores chronic inflammation as a unifying biological foundation for malignant transformation, irrespective of tissue origin (Table 1).

### 2.2. Core Drivers of the Inflammatory Microenvironment

#### 2.2.1. Persistent Activation of Inflammatory Signaling

Accumulating evidence has established chronic inflammation as a fundamental biological driver of cancer development and progression. At the molecular level, unresolved inflammatory responses lead to sustained immune activation and prolonged release of inflammatory mediators, which together reshape the tissue microenvironment into a pro-tumorigenic niche. Core signaling pathways closely associated with this process include:

- NF-κB signaling pathways

NF-κB comprises a group of transcription factors that regulate the expression of genes involved in diverse biological functions, such as immune regulation, inflammatory responses, cellular proliferation, and survival. The NF-κB signaling pathway includes two main branches: canonical and non-canonical. In the canonical pathway, inflammatory cytokines like IL-1 and TNFα initiate the cascade, activating the IKK complex. This leads to IκB degradation, releasing NF-κB to translocate into the nucleus, where it activates pro-inflammatory genes, driving persistent inflammation. The non-canonical pathway, reliant on NIK, processes p100 into p52, which partners with RelB to form a heterodimer regulating specific immune responses and fine-tuning the immune system. As a central regulator of both innate and adaptive immune responses, this pathway plays a critical role in cell fate determination. Hyperactivation of NF-κB leads to the overexpression of pro-inflammatory cytokines and growth factors, thereby promoting the formation of a tumor-supportive microenvironment and accelerating the inflammation–cancer transition. For example, in prostate cancer, aberrant NF-κB signaling promotes carcinogenesis by enhancing cell proliferation, invasion, and resistance to therapy [16]. The IL-17RA/ACT1/NF-κB axis is upregulated during the colorectal inflammation-to-cancer transformation, promoting the expression of inflammatory factors and matrix metalloproteinases (MMP7, MMP9). Qingre Huayu Jianpi prescription inhibits this axis, thereby reducing the inflammation-to-cancer transformation [17]. At the level of the immune microenvironment, upregulation of key NF-κB components (such as p50 and p100) represents a molecular hallmark of M2 macrophage polarization. In colorectal cancer, NF-κB promotes pro-tumorigenic M2 polarization through the TPX2/NF-κB/M-CSF signaling axis, whereas TPX2 silencing or inhibition of NF-κB phosphorylation and the TLR4/MyD88/NF-κB pathway markedly reduces the accumulation of M2 macrophages [18,19]. Inhibiting NF-κB signaling significantly reduces the risk of inflammation-associated tumors, reinforcing its central role in the inflammation-to-cancer transformation [20]. In colorectal cancer, inhibition of NF-κB can reverse cell cycle arrest and Cyclin D1 downregulation induced by blockade of the MAPK signaling pathway [21]. Deletion of IKKβ, a key regulator of the NF-κB pathway, in intestinal epithelial cells markedly reduces tumor incidence, whereas its deletion in myeloid cells primarily decreases tumor burden, highlighting the dual driving role of NF-κB in inflammation-associated tumorigenesis [22]. Xan-thohumol (XN) inhibits the progression of CAC by suppressing IKKβ, thereby blocking NF-κB signal transduction and reducing the expression of inflammatory factors [23]. In addition, inhibition of NF-κB signaling alleviates the chronic pro-inflammatory microenvironment and restores antitumor immune surveillance, thereby decoupling persistent infection and long-term inflammation from cancer risk, while concurrently regulating metaflammation and gut microbiota dysbiosis in colorectal cancer [24,25]. Collectively, these findings indicate that NF-κB pathway silencing can simultaneously target intrinsic malignant properties of tumor cells and remodel the tumor immune microenvironment, thereby enabling a multidimensional reversal of inflammation-to-cancer transformation.

- JAK-STAT signaling pathways

The JAK-STAT signaling pathway plays a core regulatory role in inflammation-cancer transformation, and its aberrant activation is broadly involved in the initiation and progression of multiple cancer types. By inducing STAT phosphorylation, this pathway sustains chronic inflammatory responses and drives immune dysregulation through the transcriptional activation of pro-inflammatory cytokines and inflammation-related genes, thereby promoting tumorigenesis [26,27]. Chronic activation of this pathway is closely associated with inflammation-driven cell proliferation, survival, and immune evasion, contributing to malignant progression, poor prognosis, and resistance to radiotherapy and targeted therapies in cancers such as breast, lung, and liver cancer [28]. In solid tumors, the IL-6/gp130/JAK/STAT3 axis represents a key oncogenic signaling cascade. In colorectal cancer, STAT3 activation promotes cell cycle progression and inhibits apoptosis by upregulating downstream targets including Bcl-xL and MYC, thereby accelerating tumor development [29]; Similarly, in hepatocellular carcinoma, CKLF1 enhances proliferation, migration, and resistance to apoptosis through activation of the IL-6/STAT3 pathway, further highlighting the central role of JAK–STAT signaling in inflammation-driven carcinogenesis [30]. Persistent JAK–STAT signaling remodels the tumor microenvironment by regulating immune cell differentiation, particularly macrophage polarization, leading to the establishment of a pro-carcinogenic inflammatory network [31]. In gastric cancer, tumor-derived exosomal BGN binds to the NONO protein in macrophages, promoting M2 polarization and CXCL10 expression; CXCL10 subsequently activates the JAK/STAT1 pathway in cancer cells, forming a positive feedback loop that facilitates tumor proliferation, invasion, and metastasis [32]; In colon cancer, overexpression of IKZF1 suppresses M2 macrophage polarization while promoting M1 polarization by inhibiting the JAK2/STAT5 pathway, whereas pharmacological activation of JAK–STAT signaling reverses this tumor-suppressive effect, revealing a bidirectional regulatory role of this pathway in macrophage polarization and inflammation-to-cancer transformation [33]. In hematological malignancies, acute myeloid leukemia (AML) harboring AF10 rearrangements recruits JAK1 kinase to activate inflammatory JAK–STAT signaling, thereby driving leukemogenesis and enhancing tumor cell survival, further underscoring aberrant pathway activation as a key mechanism linking inflammation to cancer development [34]. Collectively, the JAK–STAT signaling pathway functions as a core regulatory network in inflammation-to-cancer transformation by integrating inflammatory cues, shaping the immune microenvironment, and driving oncogenic gene expression.

- PI3K/AKT signaling pathways

The PI3K/AKT signaling pathway plays a critical role in inflammation-cancer transformation and has been extensively investigated in clinical and experimental studies of inflammation-associated carcinogenesis, where it functions as a key signaling axis linking chronic inflammation to tumor initiation [35]. For example, in models of alcoholic liver disease (ALD), RNF2 regulates USP7 and the PI3K/AKT pathway to modulate lipid metabolism and inflammatory responses, thereby promoting disease progression [36]. Similarly, in non-alcoholic fatty liver disease (NAFLD), puerarin flavonoids activate autophagy by inhibiting the PI3K/Akt/mTOR pathway, leading to reduced lipid accumulation and inflammation and consequent disease amelioration [37]. Aberrant activation of PI3K/AKT signaling is closely associated with the development of multiple cancer types. In hepatocellular carcinoma, BACH1 promotes the activation of this pathway by upregulating PDP1, accelerating cell proliferation and inhibiting apoptosis. Downregulation of PDP1 suppresses pathway activity and slows hepatocellular carcinoma (HCC) progression [38]. In ovarian cancer microenvironment, tumor-derived exosomal miR-205 activates this pathway by downregulating PTEN, promoting the polarization of tumor-associated macrophages (TAMs) toward the M2 phenotype, thereby driving tumor progression [39]. In models of colitis-associated colon cancer and colitis-associated colorectal cancer, abnormal or persistent activation of this pathway promotes tumor cell proliferation and survival. In squamous cell carcinomas, dysregulation of this pathway promotes tumorigenesis by controlling metabolic reprogramming, cell proliferation, and survival [40]. In gastric cancer, TTPAL cooperates with nicotinamide N-methyltransferase (NNMT) to activate PI3K/AKT signaling, thereby driving oncogenic progression [41]. The PI3K/AKT pathway also exhibits extensive crosstalk with other oncogenic and inflammatory signaling cascades [42]. It can enhance NF-κB nuclear translocation and transcriptional activity by phosphorylating NF-κB p65 (Ser536) or inhibiting its suppressor IκB. For instance, in glioblastoma, EGFR-induced activation of the PI3K/Akt/mTOR axis promotes tumor cell survival through NF-κB p65 activation [43]. Activation of NF-κB may further upregulate components of the PI3K/AKT pathway, thereby establishing a positive feedback loop. For instance, cholesterol accumulation amplifies inflammatory responses through NF-κB signaling while simultaneously promoting activation of the PI3K/AKT pathway [44]. In vascular endothelial cells, PI3K/AKT signaling cooperates with the ERK1/2 pathway to jointly regulate apoptosis and inflammatory responses. Moreover, PI3K/AKT signaling synergizes with immune checkpoint pathways such as PD-1/PD-L1, contributing to tumor immune evasion. Collectively, these findings underscore the central role of PI3K/AKT signaling as an integrative hub connecting inflammation, metabolism, immune regulation, and tumorigenesis during inflammation-to-cancer transformation. Noncoding RNAs, including miRNAs and lncRNAs, modulate carcinogenesis by targeting key components of the PI3K/AKT pathway, highlighting their potential as therapeutic targets [45].

- MAPK signaling pathways

MAPK signaling pathway plays a pivotal role in inflammation-cancer transformation and functions as a critical molecular hub linking inflammatory responses to tumorigenesis. Accumulating evidence demonstrates that persistent activation of MAPK signaling converts chronic inflammatory stimuli into oncogenic signals by regulating key transcription factors, particularly NF-κB, thereby driving tumor initiation and progression [46,47]. During macrophage activation, MAPK and NF-κB pathways form a core inflammatory signaling network that cooperatively mediates the production of pro-inflammatory cytokines, including IL-6 and TNF-α, amplifying tumor-promoting inflammation [48]. Beyond its crosstalk with NF-κB, the MAPK pathway also interacts extensively with other oncogenic signaling cascades, including PI3K/AKT, TGF-β, and Wnt pathways. This multilayered signaling network integrates inflammatory cues with proliferative, survival, and differentiation signals, ultimately governing inflammation-associated carcinogenesis [49]. The oncogenic functions of MAPK signaling have been documented across multiple cancer types. In pancreatic cancer, calreticulin promotes tumor progression by activating ERK/MAPK signaling. In triple-negative breast cancer, the long non-coding RNA LINC00665 drives malignant progression through MAPK-related pathways. In prostate cancer, MAPK cooperates with NF-κB to modulate androgen receptor signaling, thereby enhancing tumor cell survival and therapeutic resistance. Collectively, these findings highlight the context-dependent yet broadly conserved tumor-promoting role of MAPK signaling. In HCC, the lncRNA CCHE1 promotes tumor cell proliferation, inhibits apoptosis, and drives the inflammation-to-cancer transformation by activating the ERK/MAPK signaling pathway. High expression of CCHE1 is positively correlated with tumor number, size, and TNM staging in HCC patients. Knockdown of CCHE1 significantly suppresses the activity of this pathway [50]. In CRC-related research, the lncRNA LINC02320 promotes cell proliferation and metastasis by activating GRB7 transcription, which in turn activates the MAPK signaling pathway. Once activated, this pathway forms a positive feedback loop that amplifies pro-carcinogenic signals [51]. Conversely, YWHAH suppresses autophagy through negative regulation of the MAPK/ERK signaling pathway, promoting cell proliferation, migration, invasion, and inhibiting apoptosis. Its high expression is associated with poor prognosis in CRC patients [52]. Furthermore, in CRC with KRAS and BRAF mutations, inactivation of the proprotein convertase furin suppresses the activation of the ERK/MAPK signaling pathway, reducing tumor cell proliferation, migration, and invasion capabilities. Furin participates in the regulation of this pathway by activating multiple pro-oncoprotein precursors and is closely associated with the inflammation-to-cancer transformation in CRC [53]. In CAC, p38γ and p38δ, as members of the MAPK family, regulate the production of inflammatory cytokines and the recruitment of immune cells, promoting intestinal inflammation and tumorigenesis. Deletion of p38γ/δ reduces tumor formation and decreases the expression of pro-inflammatory factors [54]. In colon cancer, MK2, a key molecule in the MAPK signaling pathway, promotes tumor cell proliferation and survival by regulating the production of inflammatory factors. MK2 inhibitors reduce chemokine levels, suppress macrophage infiltration, and block the inflammation-to-cancer transformation process [55]. Additionally, studies have shown that cadmium (Cd) exposure activates the Erk/MAPK signaling pathway in normal prostate epithelial cells, promoting cell proliferation, survival, colony formation, and inhibiting apoptosis, thereby inducing malignant transformation. This pathway is significantly activated in prostate cancer tissues [56]. Aberrant MAPK activation facilitates EMT and tumor metastasis. ERK/MAPK-mediated activation of Integrin/EGFR signaling has been shown to promote EMT in pancreatic cancer, whereas pharmacological inhibition of the MAPK/ERK pathway can reverse EMT phenotypes in gastric cancer [57,58]. These findings underscore the critical role of MAPK signaling in cancer cell plasticity and metastatic dissemination. In addition to its direct effects on tumor cells, MAPK signaling reshapes the inflammatory tumor microenvironment. By regulating molecules such as CD36, MAPK contributes to metabolic reprogramming and immune modulation, thereby facilitating the transition from chronic inflammation to malignancy [59]. Moreover, non-canonical NF-κB signaling associated with MAPK activity contributes to chemotherapy resistance, further reinforcing malignant phenotypes [60]. Given its central involvement in multiple hallmarks of cancer, aberrant MAPK activation represents an attractive therapeutic target. Pharmacological inhibition of upstream kinases, such as p38, can suppress pro-tumorigenic processes including dendritic cell maturation, while combined targeting of MAPK/NF-κB signaling nodes (e.g., ERK or IκB kinase inhibition) may provide enhanced efficacy in preventing inflammation-to-cancer transformation [61].

- TGF-β/Smad signaling pathways

The pro-inflammatory mechanisms of the TGF-β/Smad signaling pathway are complex and diverse. It promotes M1 macrophage polarization via Smad-dependent and -independent pathways, upregulates inflammatory genes, and releases cytokines. It also enhances endothelial adhesion molecule and chemokine expression to recruit neutrophils. Additionally, it cooperates with transcription factors and epigenetic modifications to activate inflammatory transcription. EMT activation reshapes the inflammatory microenvironment, forming a positive feedback loop with fibrosis. In chronic inflammation, it suppresses Treg function, reduces anti-inflammatory cytokines, disrupts immune balance, and collectively drives sustained inflammation.

The TGF-β/Smad signaling pathway plays a critical role in the initiation and progression of inflammation and tumors by regulating processes such as cell proliferation, differentiation, and apoptosis. Its abnormal activation or disrupted transmission can promote the transformation from inflammation to cancer. In the inflammatory microenvironment, abnormalities in this pathway can lead to immune evasion and tumor cell proliferation. SMAD4, as a key mediator, plays a critical role in this process. Its deletion or mutation disrupts normal signal transduction, thereby promoting the development of inflammation-related cancers, such as pancreatic cancer, colorectal cancer, and cholangiocarcinoma [62]. Specifically, in digestive system diseases, abnormal TGF-β signaling promotes inflammation and cancer progression. For instance, during the transformation from gastritis to gastric cancer, elevated levels of TGF-β1 play a dual role: Helicobacter pylori infection activates the TGF-β1/Smad2 signaling pathway by upregulating HKDC1 expression, inducing the expression of EMT-related proteins and thereby driving the transformation from gastritis to gastric cancer [63]. On the other hand, it activates Smad signaling to induce EMT, promoting tumor invasion and metastasis, while simultaneously modulating immune cell function to influence the tumor microenvironment [64]. In intestinal epithelium with Smad4 deficiency, elevated TGF-β1 levels directly upregulate YAP expression and interact with Smad2/3, activating inflammation-related gene expression and exacerbating the development of colitis and colitis-associated cancer. YAP/TAZ, as downstream mediators, play a critical role in this process [65]. In cholangiocarcinoma, AMDHD1 activates the TGF-β signaling pathway by inhibiting the ubiquitin-mediated degradation of SMAD4 and enhancing the phosphorylation of SMAD2/3, thereby suppressing tumor cell proliferation and migration. Downregulation of its expression promotes tumor progression [66]. Furthermore, in mouse models with hepatocyte-specific deletion of TAK1, activation of the TGF-β signaling pathway leads to hepatocyte apoptosis, inflammation, fibrosis, and the development of HCC. Through the Smad pathway, it induces hepatocyte death and promotes compensatory proliferation while upregulating the expression of anti-apoptotic and pro-tumorigenic factors, driving HCC formation and enhancing tumor growth and angiogenesis [67]. These studies collectively demonstrate that the TGF-β/Smad signaling pathway plays a critical role in the inflammation-to-cancer transformation.

- NLRP3 inflammasome

The pro-inflammatory mechanism of the NLRP3 inflammasome is multi-step. Priming occurs when stimuli activate surface receptors, upregulating NLRP3 and pro-cytokine transcription via MyD88/NF-κB signaling. Activation is triggered by homeostatic imbalances like K^+^ efflux, mitochondrial dysfunction, or lysosomal rupture. NLRP3 then oligomerizes with ASC and NEK7 to recruit pro-caspase-1. The assembled inflammasome cleaves pro-caspase-1 into active caspase-1, which processes pro-cytokines into mature IL-1β and IL-18. Caspase-1 also induces pyroptosis, releasing inflammatory contents and creating a positive feedback loop. Excessive activation contributes to various inflammatory diseases.

The NLRP3 inflammasome plays a dual regulatory role in the inflammation-to-cancer transformation, with both its activation and inhibition significantly influencing tumor initiation and progression. In CRC, high expression of NLRP3 is associated with poor patient prognosis. It contributes to the formation of an immunosuppressive microenvironment by promoting M2 macrophage polarization and inhibiting the activation of cytotoxic CD8^+^ T cells, directly regulating both tumor cell status and immune cell function [68]. In CAC models, activation of NLRP3 drives intestinal epithelial cell proliferation, inhibits apoptosis, and disrupts barrier function by promoting Caspase-1 activation and the release of pro-inflammatory cytokines such as IL-1β, ultimately facilitating tumor formation [69]; Meanwhile, its activation can also increase the expression of matrix metalloproteinases (MMP-9/MMP-13), promoting tumor invasion and metastasis, whereas NLRP3 inhibitors can significantly inhibit this process [70]. Similarly, in CAC related to IBD, NLRP3 activation exacerbates inflammation by releasing IL-1β/IL-18, while simultaneously modulating anti-tumor immune responses, thereby providing favorable conditions for tumor growth [71]. However, the impact of NLRP3 deficiency on tumors varies. Some studies have shown that NLRP3-deficient mice exhibit reduced tumor burden, as it affects the anti-tumor function of NK cells by regulating the expansion of bone marrow-derived cells, exerting a protective effect that relies on NK cells and IFN-γ [72]. However, other studies have indicated that NLRP3-deficient mice exhibit a higher tumor burden, as it regulates the production of IL-1/IL-18 through hematopoietic cells to exert anti-tumor effects [73]. Overall, the NLRP3 inflammasome forms a dual mechanism through the release of pro-inflammatory cytokines and the regulation of immune cell functions: on one hand, it activates inflammatory responses to create a pro-cancer microenvironment; on the other hand, it weakens immune surveillance by inhibiting the activity of NK cells and CD8^+^ T cells, thereby serving as a crucial bridge linking inflammation to cancer transformation [74].

- Wnt and Hedgehog signaling pathways

Wnt and Hedgehog signaling pathways play diverse roles in inflammation. The canonical Wnt/β-catenin pathway promotes β-catenin nuclear translocation to activate inflammatory genes and modulates macrophage/dendritic cell functions. Non-canonical Wnt/PCP drives cytoskeletal reorganization and inflammatory cell migration, while Wnt/Ca^2+^ triggers inflammatory mediator release via Ca^2+^ signaling. Hedgehog signaling, through ligand-activated Gli factors, induces inflammatory gene expression, synergizes with NF-κB, regulates macrophage/T-cell activity to foster an immunosuppressive microenvironment, and can impair tissue repair when overactivated. Dysregulation of both pathways contributes to inflammatory diseases and offers potential therapeutic targets.

**Figure 1:**
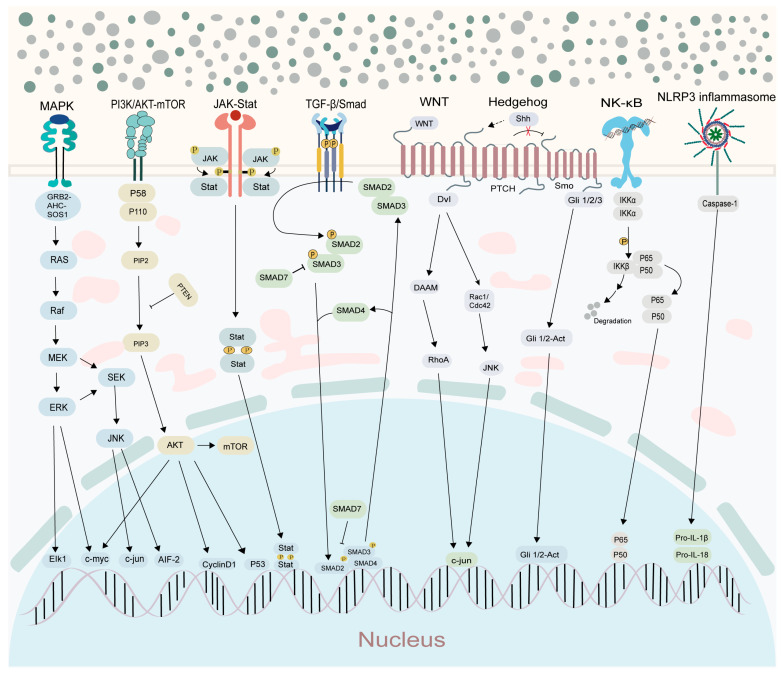
Key inflammatory and oncogenic signaling pathways involved in inflammation–cancer transformation. Chronic inflammatory stimuli activate multiple interconnected signaling cascades, including MAPK, PI3K/AKT/mTOR, JAK/STAT, TGF-β/Smad, WNT/β-catenin, Hedgehog, NF-κB, and the NLRP3 inflammasome pathways. These pathways transmit extracellular inflammatory and growth signals to the nucleus through complex phosphorylation and transcriptional regulatory networks, leading to the activation of downstream transcription factors and target genes associated with cell proliferation, survival, oxidative stress, immune dysregulation, EMT, angiogenesis, and tumor progression. Crosstalk among these pathways amplifies and sustains inflammatory signaling, thereby driving the transition from chronic inflammation to malignant transformation.

The Wnt/β-catenin signaling pathway plays a central role in the inflammatory-to-cancer transformation in the gastrointestinal tract. Its aberrant activation can drive the nuclear translocation of β-catenin, promoting the expression of downstream oncogenes (such as Ccnd1, Ccn4, Mmp7, and Myc), thereby facilitating cell proliferation, invasion, and migration. In models of CAC and ulcerative colitis-related colorectal cancer (UC-CRC), overactivation of this pathway directly promotes tumorigenesis. However, Anemoside B4 and phenolic extracts from Hulless barley grass (HBG) can significantly alleviate inflammation and block tumor formation by inhibiting the expression of key genes (Wnt6, Ctnnb1, and Myc) [75,76]. In the specific regulatory mechanism, the 5-HT7 receptor activates the Wnt pathway by interacting with CK1ε, thereby promoting the progression of CRC [77]. Meanwhile, miRNA-324-5p exacerbates pathway activation and EMT by targeting and inhibiting SUFU (a negative regulator of the Wnt pathway), thereby driving the inflammatory-to-cancer transformation in gastric cancer [78]. Meanwhile, the Hedgehog (Hh) signaling pathway also influences the inflammatory-to-cancer transformation in the gastrointestinal tract. It functions by regulating intestinal inflammation and immune responses. In patients with IBD, the activity of this pathway is reduced, and the Hh/Gli1 signaling exerts a regulatory effect on intestinal inflammation [79]. Further studies have demonstrated that the Hh pathway influences tumor growth and immune evasion by regulating the tumor microenvironment, immune cell infiltration, and the secretion of inflammatory factors, and it synergizes with the Wnt pathway to promote the development of gastrointestinal tumors [80]. In summary, the Wnt/β-catenin pathway primarily drives cell proliferation and metastasis by directly promoting the expression of oncogenes, while the Hh pathway indirectly influences tumor progression by modulating the inflammatory-immune microenvironment. Together, these two pathways constitute a critical signaling network underlying the inflammatory-to-cancer transformation (Figure 1).

#### 2.2.2. Oxidative Stress and DNA Damage

Oxidative stress is a critical driver of DNA damage within the inflammatory microenvironment and plays a central role in the initiation and progression of inflammation-related diseases, particularly cancer. Under chronic inflammatory conditions, excessive production of reactive oxygen species (ROS) disrupts redox homeostasis, leading to sustained oxidative stress and genomic instability. In prostate tissue, chronic intestinal inflammation has been shown to elevate ROS levels, thereby triggering oxidative stress responses that contribute to carcinogenesis. Excessive ROS directly attack DNA molecules, including single- or double-strand breaks and oxidative base modifications. These results result in the accumulation of oxidative DNA damage markers, including 8-hydroxy-2′-deoxyguanosine (8-OHdG) and 8-oxo-deoxyguanosine (8-oxo-dG). If not efficiently repaired, such lesions promote genetic mutations and epigenetic alterations, ultimately driving malignant transformation. Clinical evidence indicates that 8-OHdG levels are significantly higher in prostate cancer tissues than in benign counterparts, highlighting oxidative stress as a key molecular initiating event in prostate carcinogenesis [81]. Beyond direct DNA damage, persistent ROS production combined with impaired antioxidant defenses suppresses DNA repair capacity, further exacerbating genomic instability. Oxidative stress has been reported to downregulate critical DNA repair enzymes, such as Dicer and OGG1, thereby compromising damage resolution [82]. During the progression from hepatitis to cirrhosis and hepatocellular carcinoma, oxidative DNA damage markers increase markedly and correlate positively with tumor malignancy [83], underscoring the pathological significance of defective repair under chronic inflammation.

Reactive nitrogen species (RNS) also play a crucial role in inflammation-induced DNA damage. Nitric oxide (NO), predominantly generated by inducible nitric oxide synthase (iNOS), can directly inhibit DNA repair enzymes, leading to the accumulation of oxidative lesions. In chronic inflammation-associated disorders such as primary sclerosing cholangitis, iNOS upregulation coexists with elevated 8-oxodG levels. Moreover, NO interferes with the base excision repair (BER) pathway, delaying DNA repair and promoting mutagenesis and tumorigenesis [84]. Inflammatory cells further exacerbate DNA damage through the release of ROS and RNS, causing oxidative and chlorination damage. In colitis-associated carcinogenesis, ROS induced DNA damage and genomic instability activate pro-inflammatory signaling pathways, reinforcing the transition from inflammation to cancer. Concurrently, oxidative stress can modulate inflammation-related gene expression via epigenetic modifications, thereby sustaining a self-perpetuating inflammatory loop [85]. Supporting this, a Helicobacter hepaticus-infected mouse model exhibited marked accumulation of chlorinated DNA damage markers such as 5-chloro-deoxycytidine (5-Cl-dC) in colon tissue, closely associated with neutrophil infiltration. This was accompanied by downregulation of DNA repair genes involved in BER and nucleotide excision repair (NER), further aggravating genomic instability and facilitating inflammation-driven colon tumorigenesis [86]. Through the continuous generation of ROS/RNS, inflammation forms a vicious cycle of DNA damage–mutation accumulation–carcinogenesis. Certain specific factors within the inflammatory microenvironment are also involved in regulating oxidative stress and DNA damage. In the inflammatory microenvironment, ZEB1 exacerbates oxidative DNA damage by inducing the generation of ROS and pro-inflammatory cytokines. Simultaneously, ZEB1 directly suppresses the expression of the DNA repair enzyme MPG, blocking the BER pathway and leading to persistent damage. This dual action of “damage–repair inhibition” significantly increases genomic instability and promotes the malignant transformation of inflammation to colorectal cancer [87].

In summary, oxidative stress-induced DNA damage within the inflammatory microenvironment constitutes a fundamental molecular mechanism underlying the transition from inflammation to cancer. Through the combined effects of excessive ROS/RNS production, impaired DNA repair, epigenetic dysregulation, and oncogenic signaling activation, chronic inflammation drives genomic instability and malignant transformation.

#### 2.2.3. Immune Dysregulation

Under inflammatory conditions, immune cells within the tumor microenvironment (TME) undergo profound reprogramming, resulting in immune dysregulation and the establishment of an immunosuppressive niche that drives tumor initiation and progression. Among these immune components, macrophages, neutrophils, and regulatory T cells play particularly prominent roles.

Macrophages, especially TAMs, represent one of the dominant immune populations in inflammation-related tumors. In the CRC microenvironment, inflammatory cues markedly enhance TAM polarization toward the M2 phenotype. These M2-like TAMs promote tumor progression by secreting pro-tumorigenic factors such as vascular endothelial growth factor (VEGF), thereby facilitating angiogenesis, EMT, and metastatic dissemination [88]. Notably, a distinct subset of TAMs characterized by C1Q^+^TPP1^+^ expression expands significantly under inflammatory stimulation. This subset promotes CRC progression through the secretion of cytokines including IL-6 and MCP-1 and exhibits strong immunosuppressive properties that support tumor cell proliferation and invasion. Moreover, inflammatory signaling suppresses p53 activity in TAMs via SETD8-mediated methylation, further reinforcing macrophage-driven tumor progression in CRC [89]. Under inflammatory conditions such as ulcerative colitis (UC) and its associated CAC, neutrophil infiltration is markedly increased, becoming a dominant immune cell type. By releasing substances like myeloperoxidase (MPO), neutrophils promote inflammation and tumor progression [90].

Neutrophils are another key inflammatory immune cell population that undergoes functional alteration in cancer. In breast cancer, neutrophils accumulate in large numbers and acquire an immunosuppressive phenotype. These cells secrete inhibitory mediators such as IL-10, (Arg), and iNOS, which collectively suppress T-cell infiltration and function. Correspondingly, the number of CD3^+^ T cells is significantly reduced, reflecting impaired immune surveillance and contributing to tumor progression [91]. In inflammatory bowel diseases such as UC and their malignant progression to CAC, neutrophil infiltration is markedly increased and becomes a dominant immune feature. Activated neutrophils promote inflammation and tumor development through the release of myeloperoxidase (MPO) and other cytotoxic mediators.

Regulatory T cells (Tregs) and T-cell dysfunction represent another hallmark of inflammation-induced immune suppression. Tregs are a specialized subset of CD4^+^ T cells with potent immunosuppressive activity and play essential roles in maintaining immune homeostasis, preventing autoimmunity, and modulating the TME. Phenotypically, Tregs are defined by the expression of the transcription factor Foxp3 (FOXP3^+^) and high levels of CD25, the α chain of the interleukin-2 receptor. Functionally, Tregs exert immunosuppressive effects through both direct cell–cell contact mechanisms, including CTLA-4- and PD-1-mediated pathways, and the secretion of inhibitory cytokines such as IL-10 and transforming TGF-β [92]. Within the TME, Tregs are preferentially enriched in tumor tissues and facilitate immune evasion by suppressing effector T cell (Teff) activation and cytotoxicity. This immunosuppressive dominance is closely associated with tumor progression, metastasis, and resistance to therapy. For example, in breast cancer, tumor-infiltrating Tregs exhibit a highly suppressive phenotype that limits antitumor immune responses and promotes disease progression [93]. Obesity can selectively reduce the abundance of Tregs in visceral adipose tissue (VAT), thereby exacerbating metabolic inflammation. In steatotic liver disease-associated hepatocellular carcinoma (SLD-HCC), Tregs are significantly expanded and spatially clustered with cancer-associated fibroblasts (CAFs). These interactions are mediated through the TNFSF14–TNFRSF14 signaling axis, which reinforces local immunosuppression. Simultaneously, CD8^+^ T cells numbers and effector functions are markedly reduced, leading to compromised anti-tumor immunity and enhanced immune escape [94]. In addition to Tregs, other T-cell subsets contribute to inflammation-driven immune dysfunction. In liver metastatic cancer, tissue-resident iNKT17 cells emerge as key regulators of metastatic progression. These cells secrete interleukin-22 (IL-22), which acts on endothelial cells to increase vascular permeability, thereby facilitating cancer cell extravasation and liver colonization [95].

Collectively, inflammation-driven reprogramming of macrophages, neutrophils, and T-cell subsets cooperatively establishes an immunosuppressive tumor microenvironment. Through cytokine secretion, immune checkpoint modulation, metabolic reprogramming, and suppression of cytotoxic lymphocyte function, these immune alterations form a critical mechanistic link between chronic inflammation and cancer progression.

#### 2.2.4. Epigenetic Alterations

Inflammation is a key factor that induces epigenetic alterations, which play a central role in the initiation and progression of tumors. Epigenetic alterations encompass multiple aspects, including DNA methylation, histone modifications, and regulation by non-coding RNAs (such as miRNAs). These mechanisms act in concert to collectively influence gene expression patterns, thereby profoundly shaping a microenvironment conducive to tumor growth and metastasis. At the level of DNA methylation, various inflammatory factors play a crucial regulatory role. Inflammatory cytokines such as IL-6, TGFβ, TNFα, and IL-1β can activate DNA methyltransferases (DNMTs). For instance, IL-6 not only upregulates DNMT1 to promote the methylation of tumor suppressor genes but also activates the STAT3 signaling pathway to enhance the expression of DNA methyltransferases. This ultimately leads to hypermethylation and silencing of tumor suppressor gene promoter regions, while simultaneously promoting the activation of oncogenes [96,97,98,99,100,101]. In CAFs, DNA methylation patterns undergo significant alterations, characterized by global hypomethylation and hypermethylation of specific genes. These changes powerfully drive tumor growth and invasion [102]. Furthermore, during the development of CRC, inflammatory factors such as IL-6 and TNF-α also regulate DNA methylation patterns, leading to the silencing of tumor suppressor genes and the activation of oncogenes [101]. Histone modifications also constitute a crucial aspect of inflammation-induced epigenetic reprogramming. Histone-modifying enzymes such as EZH2 and LSD1 can be recruited to specific gene promoter regions, where they regulate gene expression by altering histone methylation or acetylation states. Changes such as in H3K27me3 and H3K4me3 are involved in regulating gene activation or silencing [96,97,98,99]. In CAFs, the loss of H3K27me3 contributes to their activation process, thereby influencing the expression of genes associated with tumor progression [102]. Similarly, alterations in histone modification states—such as acetylation and methylation—also participate in regulating the expression of inflammation-related genes, thereby influencing tumor cell proliferation and invasive capacity. This process is equally relevant in the development of CRC [99,101]. Non-coding RNAs also play an indispensable role in the epigenetic alterations induced by the inflammatory microenvironment. MicroRNAs such as the miR-200 and miR-34 families regulate the expression of genes related to EMT through negative feedback loops, thereby influencing the invasive and metastatic capabilities of tumor cells [97]. In CAFs, aberrant expression of miR-21 and miR-149 can further promote CAF activation and remodel the tumor microenvironment by regulating key signaling pathways [102]. Inflammatory signaling, such as the IL-6-activated STAT3 pathway, can directly regulate the expression of miRNAs like miR-21 and miR-181b-1. These miRNAs promote sustained NF-κB activation by inhibiting tumor suppressors such as PTEN and CYLD, forming a positive feedback loop that drives epigenetic alterations. Throughout this process, DNA methylation and histone modification patterns also undergo changes, collectively maintaining the transformed state of tumor cells [100]. During the transition from inflammation to CRC, miRNAs also exert significant influence on tumor progression by regulating key signaling pathways [101]. These epigenetic alterations collaborate and mutually influence one another, not only stabilizing cellular phenotypes and conferring growth advantages to tumor cells, but also promoting their proliferation, invasion, and metastasis. They accelerate the remodeling of the tumor microenvironment, serving as a key mechanism through which the inflammatory microenvironment drives the initiation and progression of cancer.

## 3. Mechanisms of Plant-Derived Compounds in Blocking the Inflammation-to-Cancer Transition

### 3.1. Suppression of Inflammation-Driven Signaling Cascades

Chronic inflammation-driven carcinogenesis is fundamentally sustained by the persistent activation of pro-inflammatory signaling cascades. These pathways serve as critical molecular bridges linking inflammatory stimuli to oncogenic transcriptional programs, thereby promoting uncontrolled cell proliferation, resistance to apoptosis, EMT, and immune evasion. Among them, NF-κB, MAPK, PI3K/Akt, and JAK/STAT pathways function as central signaling hubs and are consistently dysregulated across multiple inflammation-associated cancers. Consequently, suppression of inflammation-driven signaling cascades represents a primary and convergent mechanism by which plant-derived bioactive compounds interrupt the inflammation–cancer transition.

#### 3.1.1. NF-κB Signaling as a Core Inflammatory Hub

Accumulating evidence indicates that natural compounds can interrupt the inflammation-to-cancer transition by targeting the NF-κB signaling axis. Multiple bioactive substances exert anti-tumor effects by modulating NF-κB itself or its upstream and downstream regulatory components. At the level of upstream regulation, berberine (BBR) suppresses phosphorylation of IKKβ and p38 MAPK within the MAPK/NF-κB cascade, thereby reducing IκB degradation and NF-κB nuclear translocation, ultimately forming a barrier against malignant transformation [103]. Similarly, magnolin attenuates NF-κB activation by targeting the ERK/RSK2 and PI3K/AKT/mTOR pathways, leading to decreased IκB degradation and NF-κB nuclear accumulation [104]. The methanol extract of *Euphorbia cotinifolia* L. inhibits TAK1-mediated NF-κB/MAPK pathways while simultaneously activating the Nrf2 antioxidant pathway, synergistically suppressing IκBα degradation and NF-κB nuclear translocation [105]. Zerumbone targets MyD88-dependent NF-κB, MAPKs, and PI3K-Akt pathways, preventing NF-κB nuclear translocation by reducing IκBα phosphorylation and degradation [106]. Embelin inhibits the JNK, ERK1/2, p38, and NF-κB pathways, blocking inflammation-to-cancer progression through multiple angles [107]. Shikimic acid (SA) exerts multi-target effects by inhibiting the NF-κB and MAPK pathways, reducing inflammatory factor expression and protecting against DSS-induced ulcerative colitis-associated cancer transition [108]. Neohesperidin (NHP) synergistically inhibits the NF-κB/p65 and MAPK pathways, reducing inflammatory factor levels and tumor formation in the AOM/DSS model [109]. Several compounds act at intermediate regulatory nodes linking upstream signals to NF-κB activation. Terpenoids, for example, inhibit IKK activity, a crucial step in the NF-κB pathway, halting IκB degradation and preventing NF-κB nuclear translocation to suppress cancer transition at its source [110]. Downstream regulation of NF-κB signaling also contributes significantly to the anti-cancer effects of natural compounds. Theaflavins target the TLR4/MyD88/NF-κB pathway, reducing inflammatory responses and protecting against high-fat diet-induced cancer [111]. Luteolin, in Pseudomonas aeruginosa-induced pneumonia, inhibits the EGFR/PI3K/AKT/NF-κB and EGFR/ERK/AP-1 pathways, blocking inflammatory responses and suggesting potential in suppressing cancer transformation [112]. Galangin inhibits the NF-κB pathway, reducing inflammation and cellular proliferation to block cancer transition [113]. HD-SB (the herb pair Hedyotis diffusa-Scutellaria barbata) directly inhibits NF-κB signaling, reducing the expression of inflammatory factors and impeding the development of CAC [114]. Wogonin, in the AOM/DSS model, downregulates the expression of IL-6 and IL-1β by blocking the nuclear translocation and phosphorylation of NF-κB, thereby inhibiting cell proliferation and tumor formation [115]. In summary, these natural compounds precisely intervene in the NF-κB signaling pathway through distinct mechanisms, offering promising strategies for preventing the detrimental inflammation-to-cancer transition.

#### 3.1.2. MAPK Pathway

In chronic inflammatory conditions, aberrant MAPK activation promotes sustained epithelial proliferation, enhances EMT, and facilitates malignant transformation. Importantly, MAPK signaling often cooperates with NF-κB to amplify inflammatory and oncogenic outputs [116,117]. Accumulating evidence indicates that plant-derived compounds can attenuate MAPK signaling through distinct regulatory mechanisms. At the level of upstream regulation, the methanol extract of *Euphorbia cotinifolia* L. leaves inhibits TAK1 phosphorylation, an important upstream kinase in the MAPK pathway, subsequently blocking the MAPK signaling pathway, reducing pro-inflammatory cytokine production, and blocking inflammation-to-cancer transformation [105]. As a natural interleukin-1 receptor-associated kinase 4 (IRAK4) inhibitor, apigenin directly binds to and inhibits IRAK4 kinase activity, an upstream component that can activate downstream MAPK pathways, thereby suppressing the phosphorylation of downstream MAPK signaling pathways such as p38 and JNK, reducing pro-inflammatory factor expression, and blocking key signaling pathways involved in inflammation-to-cancer transformation [118]. In intermediate regulation, berberine inhibits the phosphorylation of p38 MAPK and ERK1/2, key kinases in the MAPK pathway, blocking lipopolysaccharide (LPS)-induced activation of the MAPK signaling pathway in microglia, reducing pro-inflammatory cytokine expression, and suppressing the MAPK pro-inflammatory signaling process driving hypothalamic inflammation-to-cancer transformation [103]. Galangin, the primary active component in *Alpinia officinarum* Hance., regulates the MAPK signaling pathway by inhibiting the phosphorylation of p38, JNK, and ERK, important kinases in the pathway, reducing inflammatory factor expression and cell proliferation, and playing an important role in blocking inflammation-to-cancer transition in various malignancies like gastric and liver cancer [113]. Hydrangenol, a key bioactive component in the standardized hot water extract (WHS) of *Hydrangea serrata*, inhibits the MAPK signaling pathway by suppressing the phosphorylation of p38 and JNK, key kinases in the pathway, reducing inflammatory factor expression and matrix metalloproteinase (MMP-13) production, and playing a significant role in blocking arthritis-related inflammation-to-joint destruction and potential cancer risks [119]. In downstream regulation, luteolin inhibits ERK phosphorylation in the epidermal growth factor receptor/extracellular signal-regulated kinase/activator protein 1 (EGFR/ERK/AP-1) signaling pathway; although ERK is a key kinase in the MAPK pathway, luteolin’s action here also affects downstream events, blocking MAPK pathway activation, reducing inflammatory factor production and M1 macrophage polarization, and playing a significant role in blocking Pseudomonas aeruginosa-induced acute pneumonia-associated inflammation-to-more severe pulmonary lesions including potential cancer risks [112].

#### 3.1.3. PI3K/Akt Pathway

The PI3K/Akt signaling pathway plays a critical role in cellular physiological activities. It regulates cell proliferation, survival, and metabolism by activating downstream effectors such as mTOR, GSK3, and NF-κB, and is also involved in inflammatory responses. However, aberrant activation of this signaling pathway can lead to the overexpression of pro-inflammatory cytokines, thereby promoting the transformation of the inflammatory microenvironment into cancer [120]. Targeting this mechanism, a variety of natural active ingredients demonstrate the potential to block the transformation from inflammation to cancer by intervening in the PI3K/Akt signaling pathway. In upstream regulation, affecting early components of the PI3K/Akt signaling pathway, eriodictyol exerts its anti-cancer effects by inhibiting the PI3K/AKT/NF-κB signaling pathway, reducing the expression of pro-inflammatory cytokines in the inflammatory microenvironment and thereby blocking the process of osteoarthritis-related inflammation transforming into cancer [121]. Quercetin also operates in a similar upstream manner by inhibiting the PI3K/Akt pro-inflammatory signaling pathway, achieving this by downregulating the phosphorylation levels of PI3K and Akt, which in turn reduces the production of inflammatory mediators and the inflammatory response, ultimately lowering the risk of cancer development [122]. The primary active components in *Scutellaria baicalensis* Georgi., such as baicalin and baicalein, share this upstream regulatory mechanism, inhibiting the PI3K/Akt pro-inflammatory signaling pathway, leading to a reduction in the production and release of inflammatory factors and thereby blocking the process of inflammation transforming into cancer [123]. Additionally, the flavonoid extract of *Polygonum viviparum* L. inhibits the PI3K/AKT pro-inflammatory signaling pathway upstream, reducing the expression of inflammatory factors and intestinal inflammation, and consequently blocking the transformation from inflammation to cancer [124]. In intermediate regulation, directly affecting key kinases or proteins in the pathway, curcumin acts at an intermediate level by inhibiting the PI3K/Akt signaling pathway, downregulating the phosphorylation levels of mTOR and p70S6K, reducing the expression of pro-inflammatory cytokines, and inducing tumor cell apoptosis, which mechanism blocks the transformation of the inflammatory microenvironment into cancer in various malignancies such as glioblastoma, breast cancer, and prostate cancer, with its specific action involving the negative regulation of the PI3K/Akt pathway to reduce the expression and activation of inflammatory factors, thereby suppressing sustained inflammatory responses and reducing cancer risk [125,126]. Formononetin (FN) also operates at this intermediate stage by reducing the levels of phosphorylated AKT and mTOR through inhibition of the pathway, blocking downstream signal transduction, suppressing tumor cell growth, and modulating related protein expression to enhance autophagy and apoptosis [127]. In downstream regulation, affecting downstream events or outcomes of the pathway activation, licorice flavonoid exhibits a unique downstream regulatory effect, although it activates the PI3K/AKT signaling pathway, it upregulates the expression of the anti-apoptotic protein Bcl-2 and downregulates the expression of the pro-apoptotic proteins Bax and caspase-3, concurrently, it inhibits the NF-κB-mediated inflammatory response and reduces the release of pro-inflammatory cytokines, thereby blocking the process of ethanol-induced gastric ulcer-related inflammation transforming into cancer [128]. Early administration of Wumei Wan (WMW) has a downstream impact by significantly inhibiting the phosphorylation levels of PI3K and AKT, blocking signal transduction to prevent the initiation and progression of CAC, indicating that WMW exerts its anti-tumor effects by regulating this pathway [129]. Furthermore, in cervical cancer research, chrysotoxine (CTX) inhibits the proliferation, migration, and invasion of cervical cancer cells by inducing ferroptosis through suppression of the PI3K/Akt pathway at a downstream level [130]. The above studies collectively indicate that inhibiting this pathway can block the progression from inflammation to cancer, and its abnormal activation is one of the key mechanisms underlying the transformation from inflammation to cancer.

#### 3.1.4. JAK-STAT Pathway

In the process of inflammation transitioning to cancer, these natural substances target various nodes within the signaling pathways, offering multiple strategies for preventing chronic inflammation from triggering cancer. For upstream regulation, which involves initial targets in the signaling cascades related to inflammation-to-cancer transformation, the ethanol extract of *Sophora flos* (SFE) and its main component, quercetin, function through multi-pathway regulation. They start by inhibiting the PI3K/Akt/mTOR signaling pathway, an early event in the inflammatory response, and also modulate the expression of proteins related to key pro-inflammatory signaling pathways such as JAK-STAT, thereby reducing the release of pro-inflammatory cytokines and blocking the process of inflammation transforming into cancer [131]. Moving to intermediate regulation, which directly affects key kinases or proteins in the JAK-STAT and related pathways, berberine specifically inhibits the activation of the JAK-STAT signaling pathway. It acts at an intermediate level by reducing the expression of inflammatory factors and the migration and invasion of tumor cells, effectively blocking the process of chronic inflammation transforming into cancer [132]. *Linderae Radix* extract (LRWE) also targets the JAK-STAT signaling pathway at this stage, reducing the expression of pro-inflammatory cytokines and suppressing colonic epithelial cell apoptosis in ulcerative colitis-related inflammation, thus blocking its transformation into cancer [133]. The 50% ethanol extract from *Allium cepa* L. (onion) peel (AP50E) directly inhibits the JAK-STAT signaling pathway, reducing the expression of pro-inflammatory cytokines and blocking the process of the inflammatory microenvironment transforming into cancer [134]. *Withania somnifera* and its primary active constituent, Withaferin A, regulate the JAK-STAT signaling pathway and further inhibit the expression of pro-inflammatory mediators such as NF-κB and COX-2, which are downstream of the JAK-STAT pathway in the inflammatory cascade, reducing the release of pro-inflammatory cytokines in the inflammatory microenvironment and blocking the process of chronic inflammation transforming into cancer [135]. Kaempferol 7-O-β-D-glucoside (KPG) inhibits the phosphorylation of JAK1/JAK2 in the JAK-STAT signaling pathway at this intermediate level, thereby blocking the activation of STAT1 (Tyr701) and STAT3 (Tyr705), reducing the expression of pro-inflammatory cytokines, and consequently blocking the process of the inflammatory microenvironment transforming into cancer [136]. For downstream regulation, which affects the final outcomes or downstream events of the pathway activation, the ethanol extract of *Alpinia galanga* (AGE) blocks the process of the inflammatory microenvironment transforming into cancer by simultaneously inhibiting the TLR4 and JAK-STAT signaling pathways. The TLR4 pathway can interact with the JAK-STAT pathway and contribute to the final release of pro-inflammatory cytokines and inflammatory mediators, which are downstream events in the inflammatory response. By reducing the release of these substances, AGE blocks the transformation from inflammation to cancer [137].

#### 3.1.5. The NLRP3 Inflammasome Pathway

The NLRP3 inflammasome is a multiprotein complex composed of the NLRP3 sensor protein, the ASC adapter protein, and the caspase-1 effector protein. It can sense various pathogen-associated molecular patterns (PAMPs) and damage-associated molecular patterns (DAMPs), and can recognize and respond to multiple stimuli. It promotes the maturation and release or maturation and secretion of the pro-inflammatory cytokines IL-1β and IL-18 by activating caspase-1 or the caspase-1 zymogen, thereby triggering inflammatory responses and playing a core regulatory and key role in the pathogenesis of various inflammatory diseases and inflammation-related diseases [138,139,140]. In the process of preventing the transition from inflammation to cancer, upstream regulation is exemplified by Oridonin (Ori), which is extracted from the traditional Chinese herb *Rabdosia rubescens* (Hemsl.) Hara. It covalently binds to the Cys279 site in the NACHT domain of the NLRP3 inflammasome, blocking the interaction between NLRP3 and NEK7 and thus inhibiting the assembly and activation of the NLRP3 inflammasome at an upstream level, playing a key role in this prevention [141]. Intermediate regulation involves multiple substances. Polyphenolic compounds in Oolong tea extract inhibit the assembly and activation of the NLRP3 inflammasome, reduce the release of inflammatory factors like IL-1β, and lower intracellular ROS levels [142]. The 85% ethanol extract of *Terminalia chebula* Retz. inhibits the activation of the NLRP3 inflammasome and the activity of its related signaling pathways (including TLR4, TLR2, NF-κB, etc.), reducing the release of inflammatory factors such as IL-1β [143]. Magnolol, the primary active component in *Magnolia oficinalis* Rehd.et Wils., inhibits the activation of the NLRP3/Caspase-1 signaling pathway, reduces the release of inflammatory factors like IL-1β, and modulates tryptophan metabolism [144]. Various diterpenoids (such as tanshinone IIA, andrographolide, oridonin) regulate NLRP3 inflammasome-related signaling pathways (including TLR4/MyD88/NF-κB, PI3K/AKT, etc.), inhibit the activation of the NLRP3 inflammasome and the release of inflammatory factors such as IL-1β [145]. These actions collectively play significant roles in blocking the transition from various types of inflammation to more severe pathological states, including potential cancer risks. Downstream regulation is represented by the 95% ethanol extract of *Antrodia cinnamomea*, which inhibits the activation of the NLRP3 inflammasome, reduces the release of inflammatory factors such as IL-1β, and alleviates endoplasmic reticulum stress. By targeting these downstream events, it plays a significant role in blocking the mechanism by which non-alcoholic steatohepatitis (NASH)-associated inflammation transitions to more severe liver lesions, including potential liver cancer risks [146] (Table 2).

### 3.2. Attenuation of Oxidative Stress and Genomic Instability

In the upstream regulation targeting oxidative stress and initial inflammatory triggers, strawberries and honey phytochemicals effectively alleviate oxidative stress and inflammatory responses by reducing ROS generation, enhancing antioxidant enzyme activity, and inhibiting the release of pro-inflammatory cytokines, thus blocking inflammation-induced carcinogenesis [147]. Green tea polyphenolic compounds scavenge excess ROS, inhibit oxidative stress responses, and downregulate pro-inflammatory cytokine expression, effectively blocking UV-induced or chronic inflammation-triggered DNA damage and cellular carcinogenesis [148]. Pomegranate, through its antioxidant properties, neutralizes ROS, inhibits oxidative stress responses, and blocks the cellular carcinogenesis pathway triggered by oxidative damage during chronic inflammation [149]. *Moringa oleifera* leaf extract active ingredients like quercetin-3-galactoside and isoquercitrin modulate oxidative stress responses and the PI3K-Akt signaling pathway, inhibiting the production of inflammatory mediators and abnormal cell proliferation to prevent the transformation of inflammation into cancer [150]. Mint active ingredients (e.g., menthol) activate the AMPK/ULK1/Nrf-2 autophagy pathway, downregulate the ERK-NF-κB and MAPK signaling pathways, and alleviate oxidative stress responses, inhibiting inflammation-induced cell carcinogenesis [151]. Arctigenin inhibits the PI3K/AKT signaling pathway and activates the AMPK signaling pathway, reducing ROS production and downregulating inflammation-related signaling pathways such as NF-κB, blocking the transformation of oxidative stress-mediated inflammation into cancer [152]. β-caryophyllene and rosmarinic acid activate the Keap1/Nrf2/ARE antioxidant signaling pathway and inhibit the NF-κB inflammatory pathway, alleviating oxidative stress responses and blocking cellular damage and carcinogenesis caused by the inflammatory microenvironment [153]. Curcumin, with its antioxidant properties, scavenges ROS, inhibits oxidative stress responses, and regulates inflammatory signaling pathways such as NF-κB, blocking chronic inflammation-induced cell carcinogenesis [154,155]. Extra virgin olive oil polyphenolic substances reduce ROS production, enhance antioxidant enzyme activity, and inhibit inflammatory signaling pathways, alleviating oxidative stress responses and blocking chronic inflammation-induced cellular DNA damage and carcinogenesis [156]. In the intermediate regulation, flavonoids exert chemopreventive effects throughout the cancer process (initiation, development, and progression). They inhibit oxidative stress, reduce DNA damage and the production of pro-inflammatory cytokines, and block the promoting effect of the chronic inflammatory microenvironment on the carcinogenic process. Additionally, by scavenging ROS and inhibiting oxidative stress responses, they block cell DNA damage and gene mutations induced by chronic inflammation [157,158]. Flavonoids and saponins, both with potent antioxidant properties, scavenge ROS, enhance antioxidant enzyme activity, and regulate inflammation-related signaling pathways such as NF-κB, thereby blocking chronic inflammation-induced cellular DNA damage and carcinogenesis [159,160]. Oolong tea phytochemicals reduce intracellular ROS levels and inhibit the activation of the NLRP3 inflammasome and the release of downstream pro-inflammatory cytokines, effectively blocking the transformation of oxidative stress-induced inflammatory responses into cancer [145]. In the downstream regulation, *Actinidia callosa var. ephippioides* polyphenolic substances, through their antioxidant activity, scavenge ROS, inhibit oxidative stress responses, and downregulate the expression of inflammatory mediators such as iNOS and COX-2, effectively blocking inflammation-induced cell carcinogenesis [161]. In summary, a variety of plant-derived bioactive compounds, such as polyphenols, flavonoids, and saponins, inhibit inflammation-induced cell carcinogenesis at the molecular level by suppressing oxidative stress and regulating inflammatory signaling pathways from upstream to downstream, demonstrating potent anti-inflammatory and anti-carcinogenic potential [147,148,149,150,151,152,153,154,155,156,157,158,159,160,161,162,163,164,165,166].

### 3.3. Epigenetic Reprogramming

Plant-derived compounds can effectively block the transformation of inflammation into cancer and reduce cancer risk through various epigenetic regulatory mechanisms. Upstream regulation involves targeting epigenetic modifications to inhibit inflammation initiation, where magnolol and the polyphenol mixture from *Magnolia officinalis*, along with sulforaphane, act by targeting histone deacetylases (HDACs). Magnolol and the *Magnolia officinalis* polyphenol mixture inhibit HDAC expression and function, increase histone acetylation levels, especially enhancing the acetylation of histone H3 in the DR5 gene promoter region, thereby activating the death receptor signaling pathway and blocking the initial steps of inflammation transforming into cancer [167]. Sulforaphane also targets HDACs, and by inhibiting their activity, it modulates histone acetylation status and non-histone target function, preventing the expression of key molecules in inflammatory signaling pathways and stopping cell malignant transformation and tumorigenesis promoted by the chronic inflammatory environment at an early stage [168]. Soy isoflavones such as genistein specifically inhibit HDAC6 activity, leading to an increase in the acetylation level of histone H3K9 and promoting the ubiquitination and degradation of the androgen receptor (AR), thus blocking the AR-mediated inflammatory signaling pathway at its initiation and effectively suppressing the initiation and development of inflammation-related cancers like prostate cancer through regulating DNA methylation and non-coding RNA expression [169]. Intermediate regulation employs multi-epigenetic modulation to interrupt inflammatory progression, with plant-derived compounds including 3,3′-Diindolylmethane, butyrate, and their derivatives acting in this stage. They inhibit HDAC activity or induce its degradation, regulate histone acetylation levels, and simultaneously modulate DNA methylation and non-coding RNA expression, further blocking the sustained activation of inflammatory signaling pathways such as NF-κB and JAK/STAT and inhibiting the secretion of pro-inflammatory factors, epigenetically interrupting the driving effect of chronic inflammation on cell malignant transformation during inflammation progression [170]. Downstream regulation involves comprehensive epigenetic control to prevent malignant transformation, where beyond HDAC regulation, plant-derived compounds like curcumin, resveratrol, and epigallocatechin gallate (EGCG) from tea act. They all inhibit the sustained activation of inflammation-related signaling pathways and block the promoting effect of the chronic inflammatory microenvironment on cell malignant transformation by regulating DNA methylation, histone acetylation/deacetylation, and non-coding RNA expression, with curcumin and resveratrol reducing the risk of various cancers [171], and EGCG and others reducing the risk of breast cancer and other cancers [172]. In summary, compounds such as curcumin, EGCG, and resveratrol inhibit the aberrant activation of inflammation-related signaling pathways and block the promoting effect of the chronic inflammatory microenvironment on cell malignant transformation through multi-layered epigenetic regulatory mechanisms from upstream to downstream, thereby reducing the risk of cancer development [173].

### 3.4. Restoration of Immune Microenvironment Homeostasis

Restoring the homeostasis of the immune microenvironment is a core strategy to block the transformation from inflammation to cancer and inhibit tumor development. This process aims to reshape the immunosuppressive microenvironment and restore immune surveillance and clearance functions through multi-dimensional regulation of immune cell functions, polarization states, and their interactions. It effectively reverses the malignant driving effect of chronic inflammation on tissue cells, thereby reducing the risk of cancer development and laying the foundation for enhancing anti-tumor immune responses.

In upstream regulation, certain natural compounds exert their effects by targeting early inflammatory signals at the initial stages, thereby preventing the occurrence of cancer. For instance, ginger-derived exosome-like nanoparticles (GELN) deliver aly-miR159a-3p to target and inhibit phospholipase C (PLC) expression in the gut microbiota, promoting docosahexaenoic acid (DHA) accumulation which suppresses c-Myc-mediated PD-L1 transcription. The reduction in tumor cell PD-L1 enhances CD8^+^ T cell infiltration and IFN-γ secretion while inhibiting M2 polarization of TAMs, ultimately blocking the transformation of chronic inflammation into melanoma [174]. In midstream regulation, certain natural compounds prevent the transition from inflammation to cancer by modulating the immune microenvironment during the progression of inflammation. In the intestinal immune microenvironment, berberine and hesperetin self-assembled nanoparticles (BBR-HST NPs) reduce pro-inflammatory cytokine expression and increase anti-inflammatory cytokine secretion in colon tissue, regulate the CD4^+^/CD8^+^ T cell subset ratio, and suppress the overactivation of γδT cells and neutrophils, restoring intestinal immune homeostasis and reducing the risk of inflammation-related cancers such as colorectal cancer [175]. Ginseng-derived exosome-like nanoparticles (GENs) modulate the intestinal immune microenvironment in two ways: by inhibiting the NF-κB signaling pathway to balance the immune microenvironment and block the progression from chronic inflammation to colorectal cancer, and further regulating it by inhibiting pro-inflammatory cytokine production and promoting anti-inflammatory cytokine expression to prevent inflammation-to-cancer transformation [176].

In the pulmonary immune microenvironment, curcumin, resveratrol, tetrahydrocurcumin, and others remodel the tumor immunosuppressive microenvironment and enhance anti-tumor immune responses by inducing immunogenic cell death (ICD), inhibiting tumor angiogenesis, regulating immune cell subsets, and modulating immune checkpoint molecule expression, reducing the risk of lung cancer [177]. In the mammary immune microenvironment, quercetin, curcumin, resveratrol, and others modulate the tumor immune microenvironment by inducing ferroptosis, promoting immunogenic cell death to activate dendritic cells and CD8^+^ T cell infiltration, and inhibiting the M2 polarization of tumor-associated macrophages and the immunosuppressive function of regulatory T cells, reducing the risk of breast cancer [178]. In downstream regulation, certain natural compounds prevent malignant transformation through comprehensive immunomodulatory effects. In the systemic immune microenvironment, resveratrol activates Sirt1 deacetylase activity, promotes p62 protein deacetylation, enhances mitophagy, inhibits mitochondrial reactive oxygen species production, reduces pro-inflammatory cytokine secretion, and regulates macrophage polarization towards the M2 phenotype, remodeling the immune microenvironment balance and reducing the risk of inflammation-related cancers [179]. The immunomodulatory peptides in Astragalus seed protein hydrolysate enhance macrophage phagocytic activity, promote ROS generation, upregulate pro-inflammatory cytokine expression, and activate the TLR4/MD2 signaling pathway, remodeling the immune microenvironment balance and reducing cancer risk [180]. The arabinogalactan from Rhodiola inhibits tumor cell migration, promotes macrophage M1 polarization and dendritic cell maturation and activation, enhances nitric oxide release and immune surface marker expression, and inhibits tumor angiogenesis, remodeling the anti-tumor immune microenvironment and blocking the malignant transformation process from chronic inflammation to cancer [181]. Baicalin and baicalein inhibit the expression of pro-inflammatory cytokines and matrix metalloproteinases by modulating the polarization and function of tumor-associated immune cells, remodeling the tumor immune microenvironment and blocking the transformation process from chronic inflammation to cancer [2]. Aloe polysaccharide ABPA1 promotes macrophage polarization towards the M2 anti-inflammatory phenotype and inhibits the release of pro-inflammatory cytokines by regulating the PI3K/Akt/GSK-3β signaling pathway, modulating the immune microenvironment and blocking the potential risk of inflammation transforming into cancer [182]. Active ingredients in *Withania somnifera* regulate the function and polarization of immune cells and inhibit the production of pro-inflammatory cytokines by modulating signaling pathways such as NF-κB and PI3K/Akt, remodeling the immune microenvironment and blocking the transformation process from chronic inflammation to cancer [183]. Flavonoids, polyphenols, and others inhibit the expression of pro-inflammatory cytokines and regulate the activation and balance of immune cells by modulating inflammatory signaling pathways such as MAPK and NF-κB, remodeling the immune microenvironment and blocking the risk of chronic inflammation transforming into cancer [184]. Polysaccharides, phenols, terpenoids, and others regulate the activity and function of immune cells and inhibit the expression of pro-inflammatory cytokines and immune checkpoint molecules, remodeling the tumor immune microenvironment and blocking the transformation process from chronic inflammation to cancer [185].

## 4. Application of Combination Therapy in Inflammation-Related Cancers

The combined application of curcumin and the COX-2 inhibitor celecoxib downregulates AP-1 and NF-κB signaling through synergistic inhibition of the COX-2/PGE2 pathway and non-COX-2-dependent mechanisms, thereby significantly enhancing growth inhibition and apoptosis induction in colorectal cancer cells. This combination provides a novel therapeutic strategy for reducing the cardiovascular toxicity of chemotherapeutic agents and improving anticancer efficacy. However, its clinical translation requires further validation of the optimal dosing ratio and long-term safety [186]. Isothiocyanates, quinones, carotenoids, and alkaloids have shown significant anti-colorectal cancer potential in combination with chemotherapy drugs or other natural compounds in laboratory studies. Through multi-target, multi-pathway synergistic effects, they enhance efficacy and reduce toxicity, providing new treatment strategies for clinical application [187]. The combination of curcumin and thymoquinone with the chemotherapeutic drug mitoxantrone (MTZ) has shown significant synergistic anti-cancer effects in breast cancer cell lines. Although the study object is breast cancer cell lines, this combination strategy also provides ideas for the treatment of cancers such as colorectal cancer, indicating its potential for translation from laboratory research to clinical application [188]. The drug delivery system formed by combining 5-fluorouracil-curcumin hybrid molecules with bacterial nanocellulose demonstrates effective inhibitory effects on colorectal cancer cells. Especially when combined with chemotherapeutic drugs or other treatment methods, it significantly improves drug targeting and bioavailability, highlighting the clinical application potential of combination therapies involving plant-derived compound drugs and other technologies [189]. In the field of breast cancer treatment, combination therapies of plant-derived active compounds have achieved fruitful results. The combined dietary intervention of the plant-derived compound sulforaphane (SFN) and the dietary fiber inulin has shown in laboratory studies that by regulating the PI3K/AKT/mTOR signaling pathway and gut microbiota composition, it significantly inhibits the growth of estrogen receptor-negative breast cancer, providing new clinical application potential for the prevention and treatment of breast cancer [190]. The combined application of quercetin and cisplatin, by inhibiting tumor cell proliferation and inducing cell apoptosis, while overcoming multidrug resistance, significantly enhances the anti-tumor effect, provides new ideas for clinical treatment, and demonstrates broad prospects from laboratory research to clinical application [191]. The combined application of resveratrol and photodynamic therapy (PDT), through synergistically enhancing cytotoxic effects and inducing tumor cell apoptosis, significantly improves the therapeutic efficacy against triple-negative breast cancer cells, provides a new effective means for clinical anti-cancer treatment, and demonstrates good application prospects [192]. The combined application of resveratrol and the chemotherapeutic drug bortezomib, through synergistic effects, significantly enhances anti-tumor efficacy, reduces cell migration ability, and regulates related gene expression, providing a new potential solution for the clinical treatment of breast cancer [193]. Curcumin-loaded nanomicelles based on quercetin-quaternized chitosan conjugates, when used in combination with the chemotherapeutic drug doxorubicin in laboratory studies, significantly enhanced the anti-cancer effect against breast cancer cells and reduced cardiotoxicity, demonstrating the clinical application potential of combination therapies involving plant-derived compound drugs and other drugs [194]. Luteolin, as a plant-derived compound, has shown significant anti-cancer effects in combination with various chemotherapeutic drugs and natural products in laboratory studies. Through multi-pathway, multi-target synergistic effects, it enhances efficacy and reduces side effects, highlighting its great potential as a combination therapy strategy in breast cancer clinical application [195]. In the treatment of other cancers and inflammation-related diseases, combination therapies of plant-derived active compounds have also yielded new discoveries. Extracts of *Acacia catechu* and *Scutellaria baicalensis*, through synergistic inhibition of NF-κB, MAPK, and PI3K-Akt signaling pathways, significantly reduce LPS-induced inflammatory factor release in alveolar epithelial cells. This combined anti-inflammatory strategy provides a new therapeutic direction for acute lung injury (ALI), and its clinical translation requires further verification of the optimal efficacy and safety of different extract ratios [196]. Ellagic acid, as a plant-derived compound, has shown significant synergistic anti-cancer effects when combined with radiotherapy and chemotherapeutic drugs such as cisplatin and doxorubicin in laboratory studies. It can enhance tumor cell sensitivity to treatment and reduce chemotherapy toxicity, demonstrating its broad prospects as a combination therapy strategy in various cancer clinical applications [197]. In inflammation-related cancers, the combination therapy of plant-derived active compounds and chemotherapeutic drugs demonstrates significant advantages. These natural compounds exert anticancer effects by regulating cancer cell events and the tumor microenvironment, characterized by low toxicity and multi-target action. When combined with chemotherapeutic drugs, they enhance efficacy, reduce drug resistance, and mitigate side effects. For example, baicalin combined with cisplatin exhibits strong synergistic effects in lung cancer [198].

## 5. Nanocarrier-Enhanced TCM for Preventing Inflammation–Cancer Transformation

TCM contains a wide spectrum of bioactive compounds that exert coordinated regulatory effects on inflammation- and tumor-related pathways. Many of these natural agents show considerable promise in interrupting the progression from chronic inflammation to cancer. However, despite their therapeutic potential, most TCM ingredients share common pharmaceutical limitations, including poor aqueous solubility, rapid metabolic degradation, and insufficient accumulation at pathological sites, which substantially restrict their in vivo efficacy.

The integration of plant-derived active compounds with nanocarriers has significantly advanced the application of TCM in preventing inflammation-driven carcinogenesis. Nanotechnology enhances the stability, bioavailability, and targeting capability of these compounds, enabling more efficient penetration into inflamed tissues and improved inhibition of carcinogenic processes. This strategy not only improves therapeutic outcomes but also reduces systemic side effects, thereby providing a solid scientific and technical foundation for the development of novel anticancer TCM formulations [199]. By encapsulating or conjugating TCM molecules within nanoscale carriers, their physicochemical properties can be favorably modified, including enhanced dispersibility, improved biological stability, and controlled or targeted drug release. Moreover, these nanosystems promote preferential accumulation in inflamed tissues or precancerous lesions, thereby strengthening the ability of TCM compounds to modulate oxidative stress, suppress pro-inflammatory signaling, and prevent cellular alterations associated with malignant transformation.

In recent years, substantial progress has been achieved in the development of nanocarrier-based TCM strategies for preventing the transition from inflammation to cancer. For example, nanoparticles loaded with *Commiphora leptophloeos* extract and engineered for colon targeting significantly reduce inflammatory markers, regulate tumorigenesis-related signaling pathways, and attenuate colon inflammation and tumor formation. In vivo studies demonstrate that these nanoparticles exhibit superior efficacy compared with the free extract while requiring lower dosages, offering a promising strategy for the prevention of IBD and CAC [200]. Ginger-derived nanoparticles (GDNPs) exhibit intrinsic colon-targeting properties and are preferentially taken up by intestinal epithelial cells and macrophages without detectable toxicity. GDNPs alleviate IBD-associated inflammatory responses, promote intestinal repair by decreasing pro-inflammatory cytokines and increasing anti-inflammatory cytokines, and significantly reduce CAC incidence and tumor growth, thereby preventing the progression from IBD to CAC [201]. Similarly, an optimized genkwanin-loaded self-nanoemulsifying drug delivery system (GKA-SNEDDS) demonstrates small droplet size, rapid drug release, and high stability, resulting in significantly improved relative bioavailability. In vivo studies confirm its ability to reduce weight loss, improve disease activity index, and inhibit colon tumor formation by inducing tumor cell apoptosis, highlighting its strong potential as an anti-CAC nanoformulation [202]. Tannic acid-containing nanoparticles prepared via turbulent mixing technology exhibit uniform particle size, high stability, and pH-responsive drug release. These nanoparticles effectively overcome the intestinal mucosal barrier, achieve colon-targeted delivery, and markedly reduce colon inflammation while exerting potent anti-tumor effects, providing new insights into nanotherapy for colon diseases [203]. In addition to epithelial-targeted strategies, nanocarriers targeting CAFs have also shown promise. As key stromal components of the tumor microenvironment, CAFs facilitate tumor metastasis through multiple mechanisms. CAF-targeted nanodelivery systems can modulate the pro-tumorigenic microenvironment, thereby inhibiting tumor cell migration, invasion, and metastasis [204]. Furthermore, nanocarrier encapsulation of TCM components such as curcumin and resveratrol enhances their ability to suppress inflammatory signaling pathways, disrupt the inflammatory microenvironment at early stages of tumor development, and prevent cancer initiation and progression by improving drug stability and tissue permeability [205]. Likewise, nanocarrier-mediated delivery of polyphenolic compounds significantly enhances their antioxidant and anti-inflammatory activities, effectively blocking inflammation–cancer transformation by improving stability and bioavailability [163]. Plant-derived nanocarriers, particularly extracellular vesicles (EVs), have emerged as versatile platforms for delivering bioactive TCM compounds. EVs can efficiently encapsulate and transport therapeutic molecules to target sites, thereby enhancing bioavailability and therapeutic efficacy. Through chemical or biological modification, EVs can achieve improved targeting and functional performance, offering innovative strategies for early intervention in inflammation-related cancers [206]. Edible plant-derived EVs have demonstrated broad applications in anti-inflammatory and anticancer therapies by protecting bioactive cargos such as siRNA, enabling targeted delivery, and effectively inhibiting inflammation and tumor growth [207].

**Figure 2:**
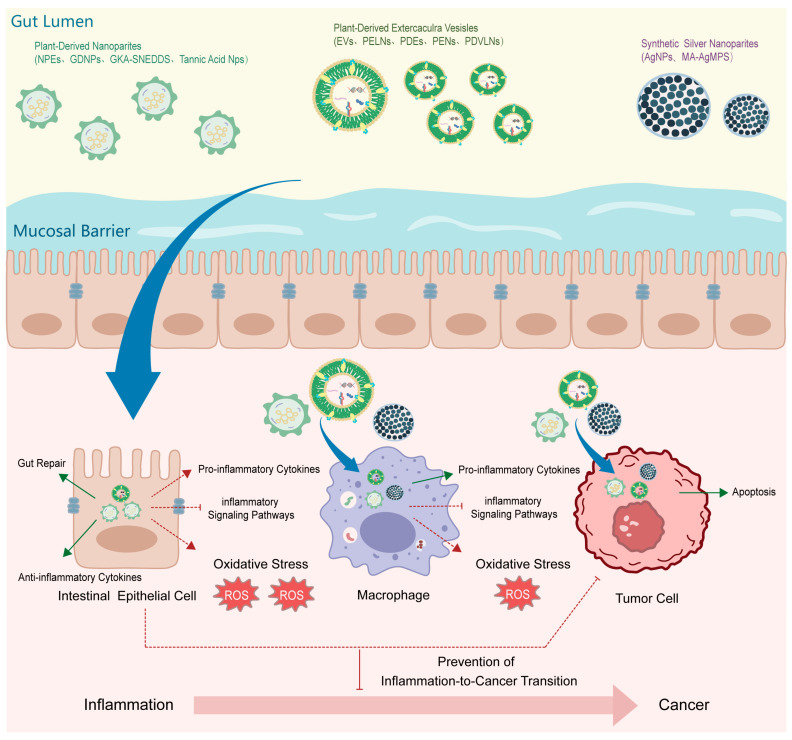
Schematic illustration of plant-derived nanomaterials in modulating gut inflammation and preventing inflammation-to-cancer transition. Plant-derived nanoparticles and extracellular vesicles as well as synthetic nanoparticles administered via the gut lumen can traverse the intestinal mucosal barrier and interact with intestinal epithelial cells, immune cells (e.g., macrophages), and tumor cells. These nanomaterials regulate inflammatory signaling pathways, reduce ROS, and modulate the balance between pro- and anti-inflammatory cytokines, thereby promoting intestinal barrier repair, suppressing chronic inflammation, and inducing apoptosis in tumor cells. Through coordinated regulation of epithelial integrity, immune responses, and oxidative stress, these nanoplatforms collectively inhibit the progression from chronic inflammation to cancer.

Plant-derived exosome-like nanoparticles (PELNs), plant-derived exosomes (PDEs), and plant exosome nanovesicles (PENs) are enriched with bioactive components and exhibit excellent biocompatibility, low immunogenicity, and intrinsic anti-inflammatory, antioxidant, and anti-tumor activities. By regulating immune responses and intercellular signaling pathways, these nanosystems effectively block the transition from inflammation to cancer while enhancing drug stability and therapeutic efficacy [208,209,210]. From a translational perspective, plant-derived vesicle-like nanoparticles (PDVLNs) demonstrate favorable biocompatibility and low toxicity, enabling efficient delivery of TCM active ingredients to target tissues. By modulating immune responses, oxidative stress, and intestinal microbiota, PDVLNs show promise in blocking the progression from IBD to CAC, although challenges remain regarding large-scale extraction and clinical application [211,212]. AgNPs prepared via green synthesis methods exhibit excellent biocompatibility and low toxicity. In preventing inflammation-cancer transformation, they can inhibit inflammation-related signaling pathways, reduce oxidative stress, and block the inflammatory microenvironment at the initial stage of cancer development. As a drug delivery carrier, it can enhance the stability and targeting of traditional Chinese medicine active ingredients, thereby strengthening their anti-cancer effects [213]. In parallel, silver nanoparticles synthesized via green or biosynthetic approaches exhibit excellent biocompatibility and anti-inflammatory properties. These nanoparticles inhibit inflammation-related signaling pathways, reduce oxidative stress, and suppress the pro-inflammatory microenvironment during early carcinogenesis. As drug carriers, they enhance the stability, targeting efficiency, and anticancer activity of TCM compounds [214] (Figure 2).

Collectively, the formulation strategies discussed above demonstrate significant potential in overcoming the bioavailability limitations of TCM. Encapsulation or conjugation with nanocarriers improves physicochemical properties, enhances biological stability, enables controlled or targeted drug release, and promotes preferential accumulation in inflamed or precancerous tissues. These advances provide promising avenues for the prevention and treatment of inflammation-associated cancers.

## 6. Future Perspectives and Recommendations

With the rapid advancement of research on plant-derived bioactive compounds and the continuous integration of multidisciplinary technologies, the development of safer and more effective anti-inflammatory and anticancer therapeutics is highly anticipated. These advances are expected not only to provide novel therapeutic options for patients with inflammation-related cancers but also to accelerate the clinical translation and application of botanical drugs. However, current research predominantly focuses on laboratory and animal models, with a significant emphasis on the still limited human clinical studies or data. This research gap substantially hinders a comprehensive understanding of the actual effects of plant-derived bioactive compounds in humans. There is an urgent need to conduct more rigorous, standardized, and large-scale human clinical studies in the future to thoroughly investigate their efficacy, safety, and mechanisms of action, thereby providing a more solid scientific foundation and practical therapeutic strategies for the prevention and treatment of inflammation-related cancers.

Future therapeutic strategies for inflammation-related cancers should prioritize a comprehensive understanding of the multi-target and multisystem mechanisms of plant-derived bioactive compounds. Particular attention should be paid to their interactions with the host immune system and the gut microbiota, both of which play pivotal roles in regulating inflammatory responses, maintaining immune homeostasis, and suppressing tumor initiation and progression [215]. It is therefore recommended to employ integrated multi-omics approaches—including transcriptomics, proteomics, metabolomics, and metagenomics—to systematically elucidate the complex molecular networks underlying these interactions. Such efforts will provide a robust theoretical basis for the rational design and optimization of plant-based therapeutic interventions.

The incorporation of artificial intelligence (AI)–assisted drug discovery technologies represent a promising strategy to significantly accelerate the development of novel anti-inflammatory and anticancer agents. Traditional screening of bioactive compounds from diverse plant sources is often time-consuming, labor-intensive, and costly. In contrast, AI-driven approaches can integrate large-scale chemical, biological, and clinical datasets to construct predictive models capable of efficiently identifying plant-derived compounds with high therapeutic potential. Moreover, machine learning algorithms can help predict molecular targets, mechanisms of action, and structure–activity relationships, thereby narrowing the screening scope and guiding subsequent drug optimization and clinical trial design.

PDEs have emerged as a promising platform in precision medicine due to their unique advantages in delivering anti-inflammatory and anticancer agents [209]. PDEs exhibit excellent biocompatibility and low immunogenicity, and they can protect encapsulated bioactive components from enzymatic degradation while enhancing in vivo stability. Nevertheless, current methodologies for PDE extraction, purification, and functional modification remain suboptimal. Future research should focus on improving isolation and purification techniques to increase yield and purity, as well as on advanced surface engineering strategies to enhance targeting specificity toward inflamed tissues or tumor sites. Such optimizations will be critical for improving bioavailability and achieving precise, efficient, and personalized therapeutic outcomes.

## 7. Conclusions

Chronic inflammation is a critical driving force in cancer initiation and progression, and inflammation–cancer transformation represents a fundamental pathological process underlying multiple malignancies. Accumulating evidence indicates that plant-derived bioactive compounds exhibit substantial potential in the prevention and treatment of inflammation-related cancers due to their multi-target and multi-pathway regulatory properties. These compounds exert anti-inflammatory and anti-tumor effects by modulating oxidative stress, inhibiting pro-inflammatory and oncogenic signaling pathways—including NF-κB, PI3K/Akt, MAPK, and JAK/STAT—regulating epigenetic modifications, restoring immune homeostasis, and remodeling the tumor microenvironment, thereby effectively interrupting the progression from chronic inflammation to malignancy. Among these regulatory networks, the PI3K/Akt and NF-κB pathways emerge as particularly promising targets for clinical translation. The PI3K/Akt pathway plays a central role in cell survival, proliferation, and metabolism, and its dysregulation is closely associated with tumorigenesis and cancer progression. Plant-derived bioactive compounds can selectively suppress aberrant activation of this pathway, thereby inhibiting tumor growth and metastasis. Similarly, the NF-κB pathway is a key mediator of inflammatory responses and is frequently hyperactivated in cancer, promoting tumor development and progression. Modulation of NF-κB signaling by these compounds effectively attenuates inflammation and restrains cancer cell proliferation. Together, these well-defined molecular mechanisms provide a solid theoretical basis for the clinical application of plant-derived bioactive compounds in inflammation-related cancers.

Despite substantial progress in preclinical studies of representative phytochemicals such as curcumin, resveratrol, and quercetin, their clinical translation remains constrained by poor bioavailability, limited stability, and insufficient clinical validation. Emerging delivery strategies, particularly plant-derived exosomes and other nanoplatforms, offer promising solutions to enhance the stability, targeting efficiency, and therapeutic efficacy of these compounds.

Among the wide range of plant-derived bioactive agents, curcumin exhibits unique advantages in blocking inflammation–cancer transformation due to its ability to simultaneously regulate multiple inflammation and cancer-related signaling pathways. Supported by extensive preclinical evidence, curcumin represents one of the most promising candidates for clinical translation. Resveratrol likewise demonstrates strong therapeutic potential by modulating immune cell function and inhibiting tumor cell proliferation and migration in various inflammation-related cancer models, positioning it as another key candidate for further clinical development.

Future research should focus on elucidating the complex interactions between plant-derived bioactive compounds, the immune system, and the gut microbiota, while fostering multidisciplinary collaboration to accelerate clinical translation. Collectively, selected plant-derived bioactive compounds have demonstrated preliminary potential as safe and effective complementary or alternative therapeutic strategies for inflammation-related cancers, providing strong support for the continued development of plant-based medicines within the framework of integrative oncology.

## Figures and Tables

**Table 1:** Inflammation-Associated Cancers.

Inflammation	Cancer	Reference
Inflammatory bowel disease	Colorectal cancer	[9]
Esophagitis	Esophageal cancer	[10]
Pancreatitis	Pancreatic cancer	[11]
Hepatitis	Liver cancer	[12]
Prostatitis	Prostate cancer	[13]
Endometritis	Endometrial cancer	[14]
Pelvic inflammatory disease	Ovarian cancer	[15]

**Table 2:** Summary of the anti-inflammatory and anti-tumor mechanisms of active ingredients in traditional Chinese medicine.

Traditional Chinese Medicine	Botanical Name	Active Ingredient	Anti-Inflammatory Mechanism	Anti-Tumor Mechanism	Reference
*Coptis chinensis* Franch., *Phellodendron amurense* Rupr.	*Coptis chinensis* Franch., *Phellodendron amurense* Rupr.	Berberine	Inhibit p38 MAPK/ERK1/2 phosphorylation and IKKβ activation, reduce NF-κB p65 nuclear translocation; regulate microglial polarization, decrease M1 markers and increase M2 markers.	–	[103]
*Magnoliae Flos*	*Magnolia biondli* Pamp.	Tetrahydrofurofuranoid Lignans	Inhibit NF-κB p65/p50 phosphorylation and nuclear translocation; suppress MAPK phosphorylation; reduce pro-inflammatory cytokine expression.	–	[104]
*Euphorbia cotinifolia* L.	*Euphorbia cotinifolia* L.	Catechin, Rutin, Quercetin	Inhibit TAK1 phosphorylation, block IκBα degradation and p65 nuclear translocation; suppress MAPK phosphorylation; reduce the production of NO and other substances.	–	[105]
*Zingiber zerumbet* (L.) Roscoe ex Sm.	*Zingiber zerumbet* (L.) Roscoe ex Sm.	Zerumbone	Inhibit TNF-α and other inflammatory products; block NF-κB activation; suppress MAPKs/Akt phosphorylation; inhibit TLR4/MyD88 expression.	–	[106]
*Ardisia elliptica*	*Ardisia japonica* (Thunb.) Blume.	Embelin, Syringic acid	Inhibit the expression of pro-inflammatory cytokines; suppress the phosphorylation of signaling pathways.	–	[107]
*Foeniculum vulgare* Mill.	*Foeniculum vulgare* Mill.	Shikimic Acid	Inhibit NF-κB P65 phosphorylation and reduce pro-inflammatory cytokine expression; suppress MAPK pathway phosphorylation and decrease inflammatory response.	–	[108]
*Ginkgo biloba* L., *Salvia miltiorrhiza* Bunge., *Tripterygium wilfordii* Hook. F., *Isodon rubescens* (Hemsl.) H. Hara., *Glycyrrhiza uralensis* Fisch., *Panax ginseng* C. A. Mey.	*Ginkgo biloba* L., *Salvia miltiorrhiza* Bunge., *Tripterygium wilfordii* Hook. F., *Isodon rubescens* (Hemsl.) H. Hara., *Glycyrrhiza uralensis* Fisch., *Panax ginseng* C. A. Mey.	Ginkgolides, Tanshinone IIA, Triptolide, Carnosol, Carnosic acid, Oridonin, Ponicidin, Glycyrrhizin, Ginsenosides	Inhibit IκBα phosphorylation and degradation, reduce NF-κB nuclear translocation and DNA binding activity, thereby blocking NF-κB system activation.	Inhibit proliferation and metastasis, induce apoptosis, suppress angiogenesis, modulate immune response and function, reduce inflammatory reactions, and block invasion and metastasis.	[110]
*Prunus zippeliana* Miq.	*Prunus zippeliana* Miq.	Theaflavins	Inhibit the NF-κB signaling pathway and reduce the release of pro-inflammatory cytokines.	–	[111]
*Alpinia officinarum* Hance.	*Alpinia officinarum* Hance.	Galangin	Inhibit the NF-κB signaling pathway and reduce the release of pro-inflammatory cytokines.	Induce cancer cell apoptosis, inhibit proliferation/migration/angiogenesis, block cell cycle, and modulate ROS to promote apoptosis.	[113]
*Citrus reticulata* Blanco.	*Citrus reticulata* Blanco.	Eriodictyol	Inhibit the activity of the PI3K/AKT, NF-κB pathway, reduce the release of inflammatory factors, and alleviate inflammatory responses in osteoarthritis.	–	[121]
*Scutellaria baicalensis* Georgi.	*Scutellaria baicalensis* Georgi.	Baicalin, Baicalein, Wogonoside, Wogonin, Oroxylin A	Inhibit TLR4 expression to reduce NF-κB nuclear translocation and inflammatory factors; block MAPK, Akt phosphorylation to decrease inflammatory mediators; activate PPAR, Nrf2 pathways to upregulate antioxidant genes.	Induce tumor cell apoptosis; inhibit tumor cell proliferation and migration; suppress tumor angiogenesis; modulate the tumor microenvironment.	[123]
*Polygonum viviparum* L.	*Polygonum viviparum* L.	Kaempferol, Luteolin, Galangin, Quercetin	Inhibit PI3K/AKT, NF-κB to reduce inflammatory factors; modulate gut microbiota to increase beneficial bacteria and decrease F/B ratio; lower uric acid to reduce inflammatory responses.	–	[124]
*Curcuma longa* L.	*Curcuma longa* L.	Curcumin	–	Inhibits PI3K/Akt pathway activity and induces tumor cell apoptosis; inhibits tumor cell proliferation, migration, and invasion; regulates autophagy and induces cell cycle arrest.	[125]
*Curcuma longa* L.	*Curcuma longa* L.	Curcumin	By inhibiting the PI3K/AKT pathway, reduce the expression of inflammatory factors; suppress the activity of inflammation-related enzymes.	Inhibit PI3K/AKT to induce tumor apoptosis; block tumor proliferation, migration, and invasion; regulate autophagy and cell cycle arrest; suppress tumor angiogenesis; enhance radiosensitivity and chemosensitivity.	[126]
*Glycyrrhiza uralensis* Fisch.	*Glycyrrhiza uralensis* Fisch.	Licorice flavonoid	Inhibit the NF-κB signaling pathway, reduce the levels of inflammatory factors; decrease oxidative stress.	–	[128]
*Sophora flos*	*Sophora japonica* L.	Rutin	Inhibit the expression of pro-inflammatory factors and promote anti-inflammatory factor expression; repair the intestinal mucosal barrier; regulate macrophage polarization; reduce oxidative stress.	–	[131]
*Coptis chinensis* Franch.	*Coptis chinensis* Franch.	Berberine	Inhibit the expression of pro-inflammatory factors; suppress inflammatory signaling pathways; regulate macrophage polarization; inhibit the NLRP3 inflammasome.	Induce tumor apoptosis; inhibit migration and invasion; block cell cycle; suppress angiogenesis; enhance anti-tumor immunity.	[132]
*Linderae Radix*	*Lindera aggregate* (Sims) Kosterm.	Linderalactone, Linderane, Lindenenol, Norisoboldine	Inhibit the expression of pro-inflammatory factors; suppress JAK-STAT pathway activation; protect the intestinal mucosal barrier; inhibit cell apoptosis.	–	[133]
*Allium cepa* L.	*Allium cepa* L.	Quercetin, Kaempferol	Inhibit JAK-STAT pathway activation; reduce the secretion of pro-inflammatory factors; suppress macrophage activation; exert antioxidant effects.	–	[134]
*Withania somnifera*	*Withania somnifera*	Withaferin A, Withanolides	Inhibit the NF-κB pathway; regulate the JAK-STAT pathway; suppress the AP-1 and MAPK pathways; activate the Nrf2/HO-1 pathway; inhibit COX-2 enzyme activity.	Induce tumor cell apoptosis; inhibit tumor cell proliferation; suppress tumor angiogenesis; reverse tumor drug resistance.	[135]
*Cudrania tricuspidata* (Carr.) Bur.	*Cudrania tricuspidata* (Carr.) Bur.	Kaempferol 7-O-β-D-glucoside	Inhibit the NF-κB pathway; suppress the AP-1 pathway; block the JAK-STAT pathway.	–	[136]
*Alpinia galanga*	*Alpinia officinarum* Hance.	Quercetin, Kaempferol, Galangin	Inhibit the TLR4, MyD88, NF-κB pathway; suppress the JAK-STAT pathway; inhibit the p38 MAPK and JNK pathways; downregulate key inflammatory enzymes.	–	[137]
*Rabdosia rubescens* (Hemsl.) Hara.	*Rabdosia rubescens* (Hemsl.) Hara.	Oridonin	Covalently binds to the Cys279 residue of NLRP3.	–	[141]
*Terminalia chebula* Retz.	*Terminalia chebula* Retz.	Tannins, Phenolic acids	Inhibit NLRP3 inflammasome activation; regulate uric acid metabolism; repair the intestinal barrier.	–	[143]
*Magnolia oficinalis* Rehd.et Wils.	*Magnolia oficinalis* Rehd.et Wils.	Magnolol	Inhibit the release of inflammatory factors; block NLRP3 inflammasome activation; suppress neutrophil migration.	–	[144]
*Antrodia cinnamomea*	–	4-acetyl-antroquinol B, antrocamol LT3, 4-acetylantrocamol LT3	Inhibit NLRP3 inflammasome activation; alleviate inflammatory responses; mitigate endoplasmic reticulum stress.	–	[146]

Notes: – indicates not reported.

## Data Availability

Data sharing is not applicable.
